# Morphological and molecular characteristics of *Homoeostrichus formosana* sp. nov. (Dictyotaceae, *Phaeophyceae*) from Taiwan

**DOI:** 10.1186/1999-3110-54-13

**Published:** 2013-08-21

**Authors:** Wei-Lung Wang, Ching-Su Lin, Wook-Jae Lee, Shao-Lun Liu

**Affiliations:** 1 Department of Biology, National Changhua University of Education, Changhua 500, Taiwan; 2 Research Group for Cosmetic Materials, Jeju Biodiversity Research Institute (JBRI) & Jeju Hi-Tech Industry Development Institute (HiDI), Jeju, 697-943 Korea; 3 Department of Life Science, Tunghai University, Taichung, 40704 Taiwan

**Keywords:** Dictyotaceae, *Homoeostrichus formosana*, Phaeophyceae, Taiwan, Zonarieae

## Abstract

**Background:**

In the marine brown macroalgae, the morphological characters are highly similar between two widely distributed genera, *Homoeostrichus* and *Zonaria* (Dictyotaceae), thereby resulting in the difficulty of exploring their hidden biodiversity. Owing to the help of the molecular tools, it is now easy for scientists to objectively describe a new species in nature. In this study, we make a description on the *Homoeostrichus formosana* sp. nov. from Taiwan, Indo-Pacific Ocean based on the morphological evidence and molecular data.

**Results:**

Our morphological observations revealed that this species has marginal row of apical cells responsible for thallus growth and the thallus with four layers of cells except the marginal regions. The cortical cell lies upon each medullary cell in transverse section, and two cortical cells upon each medullary cell in longitudinal section. Tetrasporangium is developed from cortical cell with stalk cell and singly scattered over the thallus surface, and has no indusia and paraphyses. Molecularly, the phylogenetic trees based on *SSU*, *psaA, psbA,* and *rbcL* gene sequences supported that *Homoeostrichus* species are closely related to *Exallosorus* species and clearly separated from each others in addition to *Zonaria* species.

**Conclusions:**

*Homoeostrichus formosana* sp. nov. can now be clearly distinguished from *E. harveyanus* and Japanese *H. flabellatus*.

**Electronic supplementary material:**

The online version of this article (doi:10.1186/1999-3110-54-13) contains supplementary material, which is available to authorized users.

## Background

Phillips The three genera, *Exallosorus*^1997^ J. Agardh , Homoeostrichus 1894 C. Agardh and *Zonaria*^1817^ were established based on the characteristics of their reproductive structures, which used as key characters in the taxonomy of Dictyotaceae (Papenfuss 1944; Womersley 1987; Phillips and Clayton 1993,1994,1997; Phillips et al. 1994; Phillips 1997). Genus *Homoeostrichus* was established to include *Zonaria canaliculata* J. Agardh, *Z. multifida* Harvey *ex* J. Agardh, *Z. sinclairii* Hooker *et* Harvey and *Z. stuposa* R. Brown *ex* J. Agardh (J. Agardh 1894). Genus *Zonaria* had included five sections with *ca.* 50 species (C. Agardh 1817see Silva 1952), of which several species were transferred to *Dictyota* Lamouroux and *Padina* Adanson. Ten species of *Zonaria* are currently recognized (Phillips 1997; Phillips and Nelson 1998), and most of them are endemic to Australia (Womersley 1987; Phillips 1997; Phillips and Nelson 1998), whereas *Z. diesingiana* J. Agardh and *Z. tournefortii* (Lamouroux) Montagne are widely distributed from subtropical to temperate waters (Børgesen 1926; Taylor 1960; Gayral 1966; Allender and Kraft 1983; Seagarief 1984; Yoshida et al. 1985; Silva et al. 1987,1996; Womersley 1987; Farrant and King 1989; Ribera et al. 1992; Phillips et al. 1994; Phillips 1997; Phillips and Clayton 1997; Yoshida 1998).

Papenfuss (1944) suggested that *Homoeostrichus* and *Zonaria* shared characteristics in vegetative morphology and subsumed *Homoeostrichus* in *Zonaria*. However, Womersley (1987) argued that species of *Zonaria* had octosporangia and paraphyses whereas species of *Homoeostrichus* had only tetrasporangia and no paraphyses. He kept distinguishing *Homoeostrichus* from *Zonaria* and recognized three species of *Homoeostrichus* (*H. canaliculatus* J. Agardh, *H. olsenii* Womersley and *H. sinclairii* (Hooker *et* Harvey) J. Agardh). Phillips (1997) established *Exallosorus* based on two Australian species, *Zonaria harveyana* (Pappe *ex* Kützing) Areschoug (as *Homoeostrichus multifidus* J. Agardh) and *Homoeostrichus olsenii* Womersley [as *E. harveyanus* (Pappe *ex* Kützing) Phillips and *E. olsenii* (Womersley) Phillips]. She suggested that these species of *Exallosorus* have tetraspornagia with a stalk cell and within the indusiate sori which lack paraphyses and mucilage. The plants of genus *Homoeostrichus* commonly distributed in southeastern Australia and currently are recognized as two species: *H. canaliculatus* (Womersley and *H. sinclairii*^1987^; Phillips 1997).

**Figure 1:**
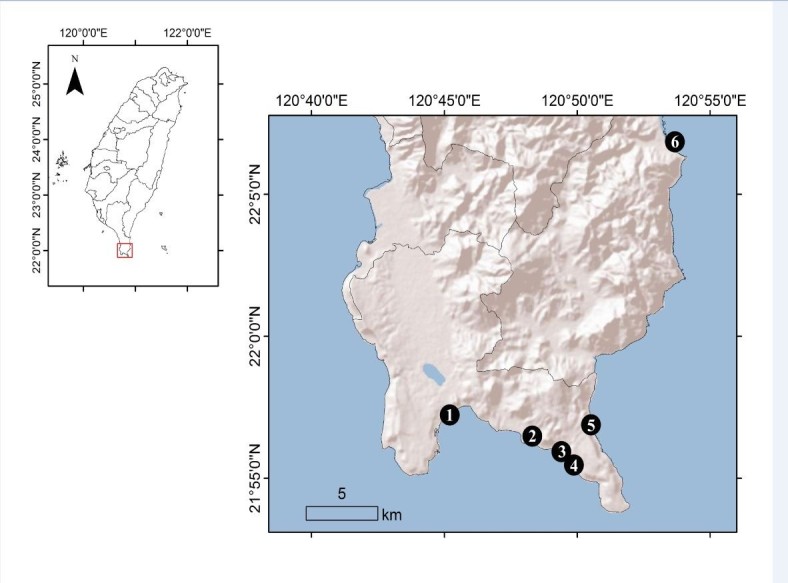
**Collection sites (Points) in southern Taiwan.** 1. Chu-Shui-Kou; 2. Chuan-Fan-Shih; 3. Hsiao-Wan; 4. Hsiang-Chiao-Wan; 5. Feng-Chui-Sha; 6. Chiu-Peng.

A species of brown alga with external morphology similar to *Exallosorus* and *Zonaria* was collected from several collecting sites (Figure 1) in southern Taiwan. The plants of *Homoeostrichus formosana* Wang, Lin, Lee *et* Liu *sp. nov.* have been identified as *Z. diesingiana* or *Z. harveyana* in Taiwan, due to short information of their reproductive structures and morphological characteristics, especially no gametangia. It is the first time to describe the characteristics of sporangia of *H. formosana* sp. nov*.* in this study. We also described the morphological and phenological characteristics of this species, and determined its phylogeny among the related species based on nuclear-encoded SSU rRNA and plastid encoded *rbcL, psaA,* and *psbA* gene sequences.

## Methods

### Survey on morphological characteristics

Collections were made by SCUBA or snorkeling in southern Taiwan (Figure 1) from 1999 to 2002. Voucher specimens were fixed with 10% formalin/sea water or pressed on herbarium sheets and deposited in the Herbarium of the Department of Biology, National Chunghua University of Education, Taiwan. Microscopic sections were made using a freezing microtome (Leica CM1850), then stained with 0.1% Toluidine Blue O (TBO) and mounted in 50% Karo syrup. Microphotographs were taken on a Pixera digital camera attached to a Carl Zeiss Axioskop 2 microscope with differential interference contrast (DIC) optics.

Other specimens deposited in the following institutions were also examined: the Institute of Oceanography, National Taiwan University, Taipei (IONTU), the National Museum of Natural Science, Taichung, Taiwan (NMNS) and the National Museum of Marine Biology and Aquarium, Hengchun, Taiwan (NMMBA).

### Gene sequence analyses

Collections for gene sequencing were made by SCUBA or snorkeling at Kenting, in southern Taiwan on 23 April 2004. Nuclear-encoded *SSU* rRNA and plastid encoded *rbcL* gene were selected for elucidating the phylogenetic relationship of *Homoeostrichus formosana* sp. nov. with other Dictyotaceae. Genomic DNA was extracted from 0.01 g of powder ground in liquid nitrogen using Dneasy Plant Mini Kit™ (Qiagen, Hilden in Germany), according to the manufacturer’s instructions. The partial *rbcL* gene and *rbcS*, except for short 3′-terminal of *rbcL* and 5′-terminal region of the *rbcS,* were amplified and sequenced as two fragments using the primers sets, DRL1F-DRL2R and DRL2F-RU2 (Hwang et al. 2005). The *psaA* gene sequences were also amplified and sequenced by two 130 F-970R and 870 F-1760R primers sets, *psbA* gene by one fragment with psbA F- psbA R primers set (Yoon et al. 2002). The partial 18S rRNA gene (*SSU*) was amplified and sequenced using primers set, SR1-SR7 and SR4-SR12. The amplified DNA was purified using High Pure PCR Product Purification Kit™ (Roche, Indianapolis,USA), in accordance with the manufacturer’s instructions. The forward and the reverse sequences were determined for all samples using an ABI PRISM 377 DNA sequencer. The sequences were aligned using PHYDIT (Chun 1995) with final visual confirmation and then submitted to GenBank under the accession numbers (Table 1). The alignment of each coding gene sequence was based on the alignment of inferred amino acid sequences, and reconfirmed by eye. The *Padina* species were selected as the outgroup species in the phylogenetic analyses.

**Table 1:** **The list of materials and accession number of nucleotide sequences determined and used in these analyses**

Species name	Collection site & reference	GenBank accession number			
		SSU	***rbc*** L	***psaA***	***psbA***
*Dictyopteris divaricata* (Okamura) Okamura	Atsumi, Yamagata, Japan; 28.iv.2002 (Hoshina et al. 2004)	AB087112	-	-	-
	Jindo, Jeolanamdo, Korea; 20.vii.1998, coll. W.J. Lee (Hwang et al. 2004)	-	AY430322	AY430305	AY430342
	Anin, Gangwondo, Korea; 19.vi.2000, coll. I.K. Hwang (Hwang et al. 2004)	-	AY422676	AY422600	AY422638
	Haegumgang, Gyoungsangnamdo, Korea; 14.vii.2000, coll. I.K. Hwang (Hwang et al. 2004)	-	AY430328	AY430310	AY430347
*Dic. pacifica* (Yendo) I.K. Hwang, H.S. Kim & W.J. Lee	Gampo, Gyoungsangnamdo, Korea; 20.xi.2002, coll. W.J. Lee (Hwang et al. 2004)	-	AY430337	AY430315	AY430356
*Dic. polypodioides* (A.P. De Candolle) J.V. Lamouroux	Miyako Is., Okinawa, Japan; 8.v.2001 (Hoshina et al. 2004)	AB087113	-	-	-
*Dic. prolifera* (Okamura) Okamura	Tsumekizaki, Shizuoka, Japan; 19.iii.2002 (Hoshina et al. 2004)	AB095294	-	-	-
*Distromium decumbens* (Okamura) Levring	Tobishima Is., Yamagata, Japan; 13.ix.2001 (Hoshina et al. 2004)	AB087116	-	-	-
	Ullreungdo Is., Gyungsangbukdo, Korea; 15.viii.1995, coll. W.J. Lee (Lee and Bae 2002)	AF350231	AF353375	-	-
	Guryoungpo, Gyoungsangbukdo, Korea; 23.viii.1996, coll. I.K. Hwang (Hwang et al. 2004)	-	AY422683	AY422607	AY422645
*Exallosorus harveyanus* (Pappe ex Kützing) J.A. Phillips	Boulders Beach, Cape Town, South Africa; 6.ii.2005, coll. S.M. Boo (This study)	-	-	-	DQ866941
*E. olsenii* (Womersley) J.A. Phillips	Nora Creina Bay, NSW, Australia; 17.iv.2003, coll. W.J. Lee & E.C. Yang (This study)	DQ866939	DQ866923	DQ866957	-
*Homoeostrichus canaliculatus* (J. Agardh) J. Agardh	Nora Creina Bay, NSW, Australia; 17.iv.2003, coll. W.J. Lee & E.C. Yang (This study)	-	DQ866922	DQ866956	DQ866943
*H. flabellatus* Okamura	Irabu Is., Okinawa, Japan; 6.viii.2001 (Hoshina et al. 2004)	AB087118	AB096895	-	-
*H. formosana* W.L. Wang, C.S. Lin, W.J. Lee & S.L. Liu sp. nov.	Sail Rock, Kenting National Park, Taiwan; 27.iii.2004, coll. W.J. Lee & I.-K. Hwang (This study)	DQ866938	DQ866929	DQ866962	DQ866951
	Big Bay, Kenting National Park, Taiwan; 28.Mar.2004, coll. W.J. Lee & I.-K. Hwang (This study)	-	DQ866931	DQ866964	DQ866952
*H. sinclairii* (J.D. Hooker & Harvey) J. Agardh	Nora Creina Bay, NSW, Australia; 17.iv.2003, coll. W.J. Lee & E.C. Yang (This study)	-	DQ866934	DQ866968	DQ866953
	Nora Creina Bay, NSW, Australia; 17.iv.2003, coll. W.J. Lee & E.C. Yang (This study)	-	DQ866935	DQ866969	DQ866954
	Marado, Jeju Is., Korea; 1.vi.2000, coll. E.Y. Lee & I.L. Lee (Lee et al. 2003)	AY232600	-	-	-
*Lobophora pachyventera* Z. Sun, P.-E. Lim, J. Tanaka & H. Kawai	Wanlitung, Kenting National Park, Taiwan; 27.iii.2004, coll. W.J. Lee & I.-K. Hwang (This study)	-	DQ866930	DQ866963	DQ866942
*Lobophora variegata* (Lamouroux) Womersley	Malakal Is., Palau; 17.xi.2001 (Hoshina et al. 2004)	AB087119	-	-	-
	Bablomekang Is., Palau; 14.xi.2001 (Hoshina et al. 2004)	AB096086	-	-	-
	Neilson Park, Sydney, Australia; 21.x.2000, coll. Zucarello (Lee and Bae 2002)	AF350232	-	-	-
*Lobophora australis* Z. Sun, F. C. Gurgel & H. Kawai	Sea View, SA, Australia; 14.iv.2003, coll. W.J. Lee & E.C. Yang (This study)	-	DQ866924	DQ866958	DQ866944
*Padina arborescens* Holmes	Jukbyun, Gangwondo, Korea; 18 Dec. 2002, coll. I.K. Hwang (This study)	-	-	AY430316	AY430357
	Sado Is., Niigata, Japan; 1.viii.1999 (Hoshina et al. 2004)	AB087122	AB096900	-	-
*P. australis* Hauck	Henoko, Okinawa, Japan; 27.vii.2001 (Hoshina et al. 2004)	AB087123	-	-	-
*P. crassa* Yamada	Ishigaki Is., Japan; 21.i.1998, coll. W.J. Lee & J. H. Oak (Lee and Bae 2002)	AF350240	-	-	AY422643
*P. japonica* Yamada	Gyokpo, Jeolabukdo, Korea; 11.Aug.1998, coll. I.-K. Hwang (This study)	-	AB096905	AY430319	AY430360
*Spatoglossum crassum* J. Tanaka	Anin, Gangwondo, Korea; 23.xii.1998, coll. I.K. Hwang (Lee and Bae 2002; Hwang et al. 2004)	AF350222	AY430336	AY430314	AY430355
	Yumigahama, Shizuoka, Japan; 18.iii.2002 (Hoshina et al. 2004)	AB087129	AB096909	-	-
*Stypopodium flabelliforme* Weber-van Bosse	Alona Beach, Panglao Is., Bohol, the Philippines; 17.xii.2003, coll. W.J. Lee (This study)	-	DQ866928	DQ866959	DQ866947
	Pratas Is., South China Sea; 22.iv.2004, coll. S.M. Lin (This study)	-	DQ866927	DQ866960	DQ866949
*St. schimperi* (Kützing) M.Verlaque	Lebanon, France; 1.v.2005, coll. G. Bitra (This study)	-	DQ866926	DQ866961	DQ866948
*St. zonale* (J.V. Lamouroux) Papenfuss	Okiakime Is., Kahoshima, Japan; 4.ix.1999 (Hoshina et al. 2004)	AB087133	-	-	-
*Zonaria angustata* C. Agardh	Emubay, Kanggaroo Is., Australia; 15.iv.2003, coll. W.J. Lee & E.C. Yang (This study)	-	DQ866932	DQ866966	DQ866946
*Z. crenata* J. Agardh	Emubay, Kanggaroo Is., Australia; 15.iv.2003, coll. W.J. Lee & E.C. Yang (This study)	DQ866937	-	DQ866965	DQ866945
	Nora Creina Bay, New South Wales, Australia; 17.iv.2003, coll. W.J. Lee & E.C. Yang (This study)	-	DQ866933	DQ866967	DQ866950
	Nora Creina Bay, New South Wales, Australia; 17.iv.2003, coll. W.J. Lee & E.C. Yang (This study)	-	DQ866936	DQ866970	DQ866955
	Tamarama Beach, Sydney, Australia; 19.ii.1996, coll. W.J. Lee (Lee and Bae 2002)	AF350234	AF353377	-	-
*Z. desingiana* J. Agardh	Ishigaki Is., Japan; 21.i.1998, coll. W.J. Lee & J.H. Oak (Lee and Bae 2002)	AF350233	AF353378	-	-
	Seongsan, Korea; 15.viii.2001, coll. W.J. Lee (Hwang et al. 2005)	-	AY422682	AY422606	AY422644

*Abbreviation*: *CCMP* Provasoli-Guillard National Center for Culture of Marine Phytoplankton.

Phylogenetic analysis was conducted using the software MEGA with a maximum likelihood method (, Tamura *et al.*^2011^). Prior to the phylogenetic analysis, the best fit of nucleotide evolutionary model for each gene was selected based on maximum-likelihood model fitting in the software MEGA. The chosen model is TN93+*G* model for *SSU* [*lnL* = -4717.63, rates of nucleotide changes (AT: 0.05, AC: 0.04, AG: 0.08, TA: 0.05, TC: 0.20, TG: 0.06, CA: 0.05, CT: 0.25, CG: 0.06, GA: 0.07, GT: 0.05, GC: 0.04), *G* = 0.08, and nucleotide frequencies (A: 0.24, T: 0.26, C: 0.22, G: 0.28)], GTR+*G* model for *rbc* L [*lnL* = -8507.61, rates of nucleotide changes (AT: 0.12, AC: 0.02, AG: 0.09, TA: 0.11, TC: 0.13, TG: 0.03, CA: 0.04, CT: 0.27, CG: 0.02, GA: 0.12, GT: 0.04, GC: 0.02), *G* = 0.22, and nucleotide frequencies (A: 0.30, T: 0.32, C: 0.16, G: 0.22)], TN93+*G*+*I* model for *psaA* [*lnL* = -10500.23, rates of nucleotide changes (AT: 0.06, AC: 0.03, AG: 0.07, TA: 0.05, TC: 0.15, TG: 0.03, CA: 0.05, CT: 0.33, CG: 0.03, GA: 0.12, GT: 0.06, GC: 0.03), *I* = 0.47, *G* = 0.53, and nucleotide frequencies (A: 0.30, T: 0.36, C: 0.16, G: 0.19)], and GTR+*G* model for *psbA* [*lnL* = -4454.15, rates of nucleotide changes (AT: 0.15, AC: 0.01, AG: 0.06, TA: 0.11, TC: 0.17, TG: 0.02, CA: 0.01, CT: 0.37, CG: 0.004, GA: 0.07, GT: 0.03, GC: 0.003), *G* = 0.17, and nucleotide frequencies (A: 0.26, T: 0.36, C: 0.17, G: 0.21)]. The ML bootstrap analyses were conducted with 500 replicates because of high computational demands.

## Results

### Species description

#### Homoeostrichus formosana

W.-L. Wang, C.-S. Lin, W.-J. Lee & S.-L. Liu sp. nov. (Figures 2, 3, 4, 5, 6, 7, 8, 9, 10, 11, 12 and 13)

**Figure 2:**
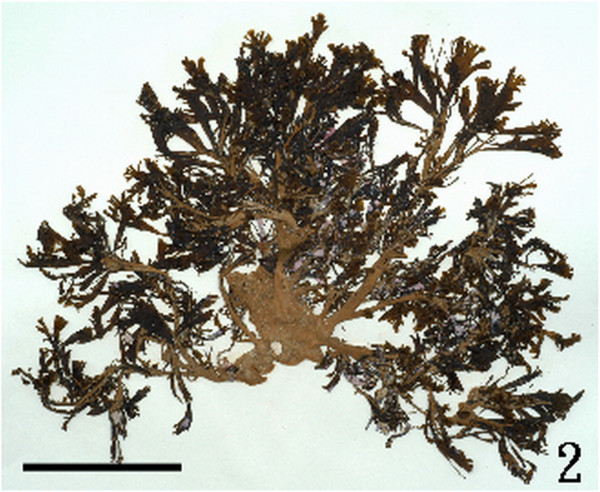
***Homoeostrichus formosana*** **sp. nov. Mature thallus, Holotype (Scale bar: 10 cm).**

**Figure 3:**
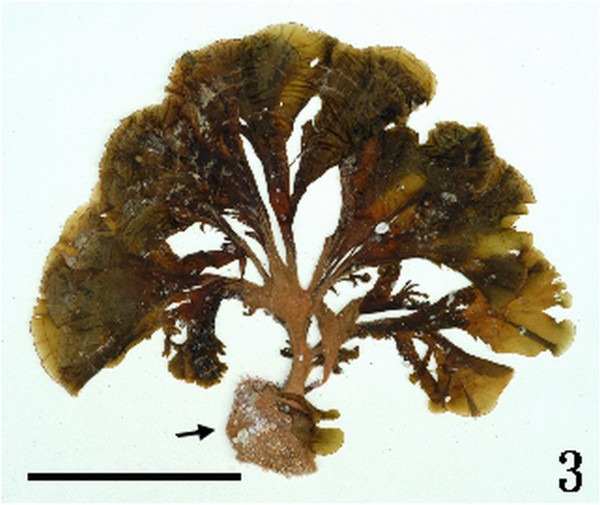
***Homoeostrichus formosana*** **sp. nov. Young thallus, with an enlarged holdfast (arrow) at the base (Scale bar: 5 cm).**

**Figure 4:**
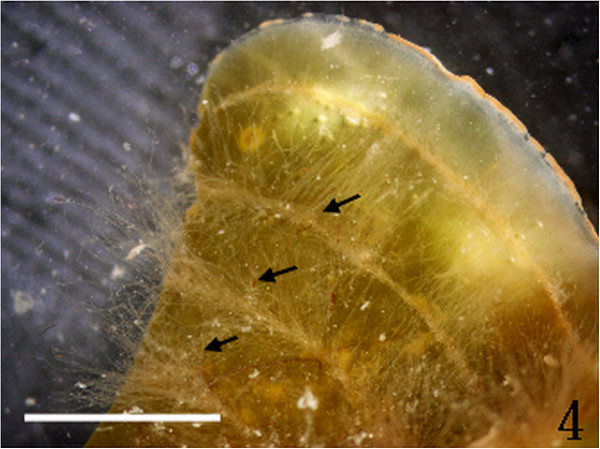
***Homoeostrichus formosana*** **sp. nov. White hairs (arrow) arranged in concentric bands (Scale bar: 2 cm).**

**Figure 5:**
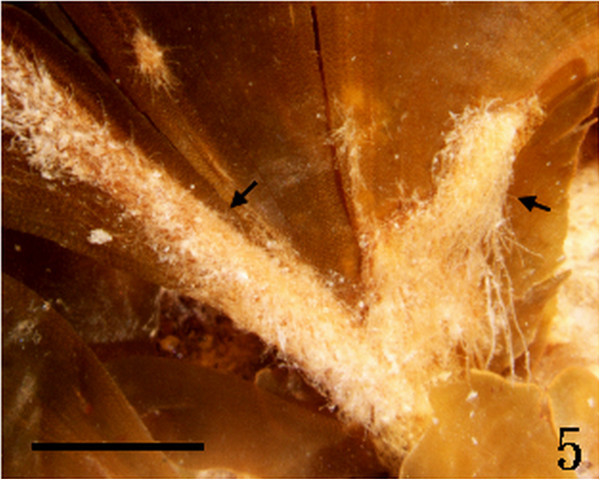
***Homoeostrichus formosana*** **sp. nov. Brown rhizoidal filament (arrow) covering over the base of thallus (Scale bar: 0.5 cm).**

**Figure 6:**
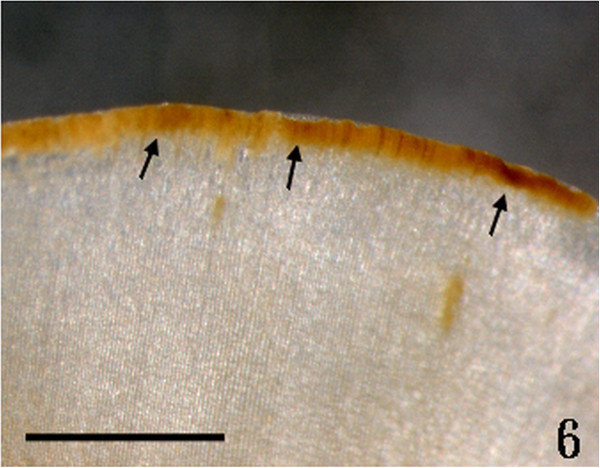
***Homoeostrichus formosana*** **sp. nov. Darker apical cell row (arrow) arranged at the thallus margin (Scale bar: 0.4 cm).**

**Figure 7:**
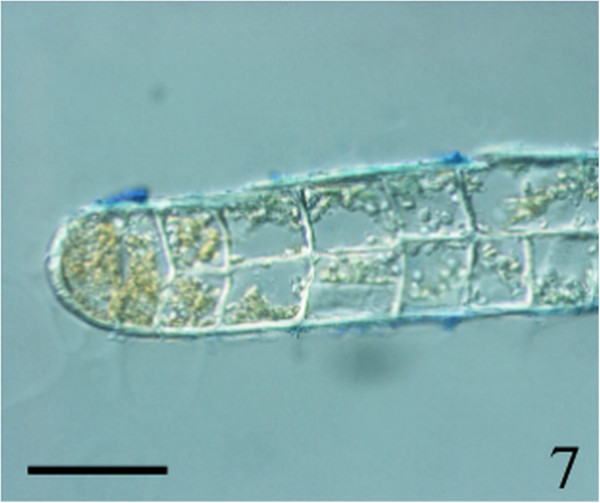
***Homoeostrichus formosana*** **sp. nov. Transverse section at the thallus margin, 2 cells-layers in thickness (Scale bar: 50 μm).**

**Figure 8:**
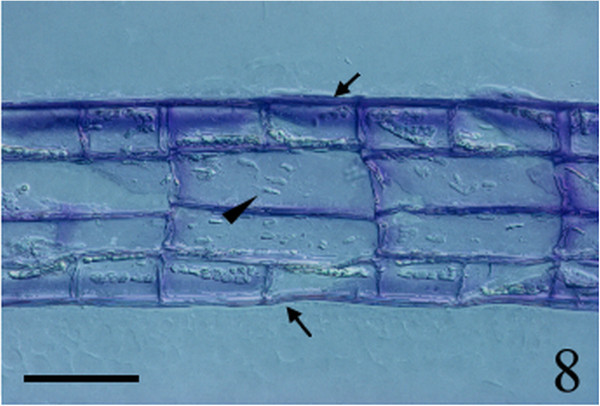
***Homoeostrichus formosana*** **sp. nov. Longitudinal section, 2–3 cortical cells (arrows) overlay each medullary cell (arrow head) (Scale bar: 50 μm).**

**Figure 9:**
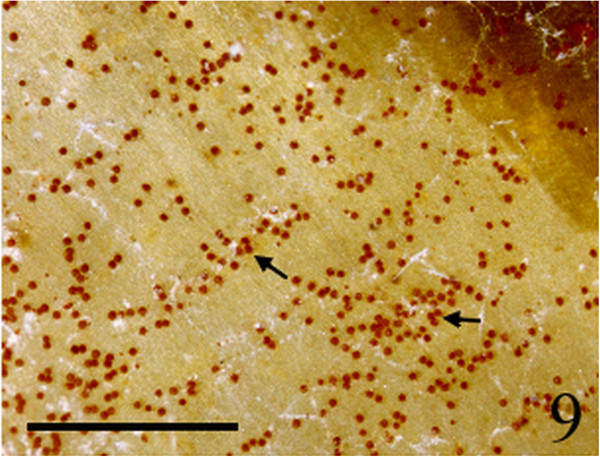
***Homoeostrichus formosana*** **sp. nov. Sporangia (arrow) scattered over a thallus surface (Scale bar: 2 mm).**

**Figure 10:**
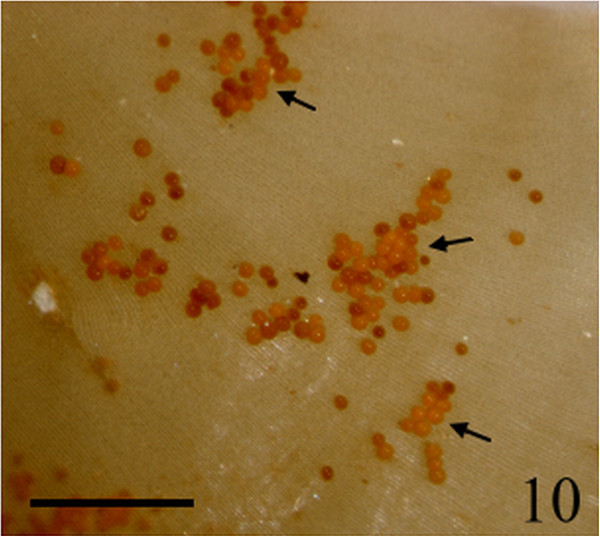
***Homoeostrichus formosana*** **sp. nov. Sporangia (arrow) scattered over a thallus surface (Scale bar: 500 μm).**

**Figure 11:**
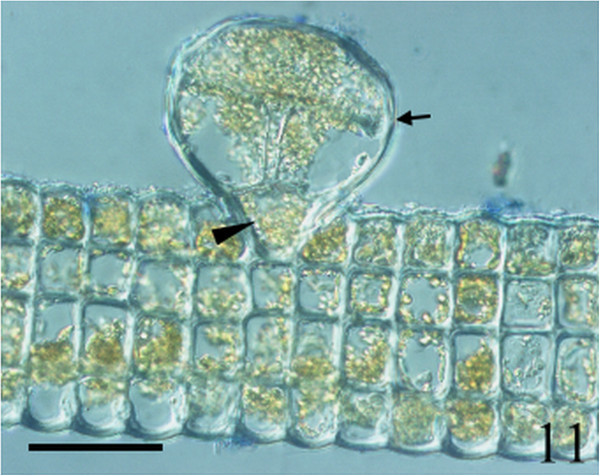
***Homoeostrichus formosana*** **sp. nov. Transverse section, showing all cells equally sized, and tetrasporangia (arrow) with a basal stalk cell (arrow head) projected above the cortex (Scale bar: 50 μm).**

**Figure 12:**
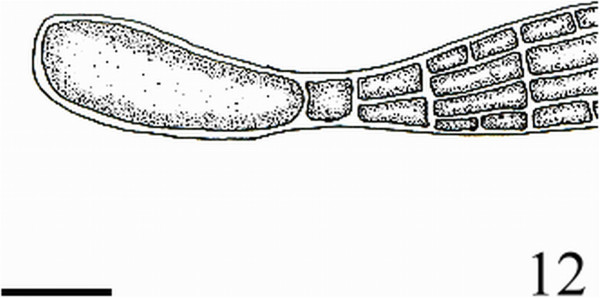
***Homoeostrichus formosana*** **sp. nov. Longitudinal section, showing an obviously apical cell (Scale bar: 80 μm).**

**Figure 13:**
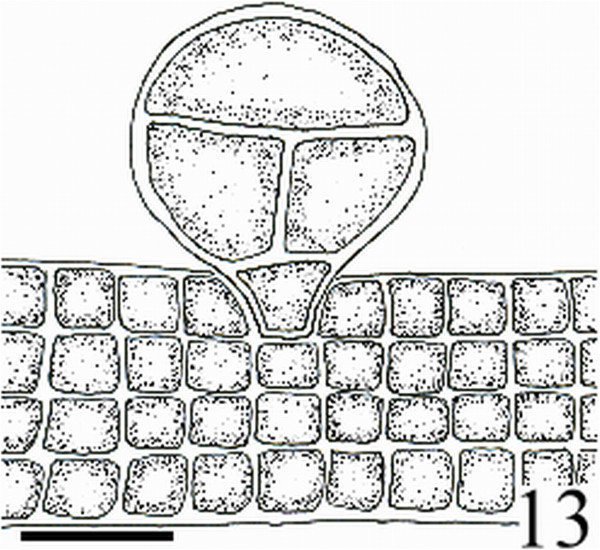
***Homoeostrichus formosana*** **sp. nov. Transverse section, showing tetrasporangia with a basal stalk cell projected above the cortex (Scale bar: 45 μm).**

*Huius plantae thallus, qui mensuratur 5–23 cm altitudine ac (1-)3-7(-10) cm latitudine, est fuscus, planus ac flabellatus; in ramos dividitur quorum axes inferiors angusti, superiors vero segmentati atque flabellate sunt. Folia in basi sunt erecta aut plana atque exsurgunt e stipite manifeste rhizoidali. Thallus autem componitur ex duo aut quattuor cellularum ordinibus, crassitudine 88–100 μm. In transversali sectione, medullares cellulae, 80–157 μm altitudine ac 15–25 μm latitudine, conteguntur a singulari cellula corticali, cui mensuratio est 25–50 μm altitudine ac 15–25 μm crassitudine. In sectione autem longitudinali, duo tresve corticales cellulae contegunt singulariam cellulam medularem. Tetrasporangia sphaerica, dispersa supra superficies, marginibus exceptis, mensuram habent 80–100 μm altitudine ac 85–95 μm in diametro, cum singularia cellula basilari quae se protrudit ultra thalli superficiem, sed sine ullis excrescentiis sori, indusii aut paraphysis.*

Thalli are 5–23 cm in height and (1-)3-7(-10) cm in width, dark brown in color, complanate, flabellate, split to form branches with narrow lower axes and upper flabellate segment, and prostrate at the base arising from a conspicuously rhizoid holdfast to upright blades. Thallus composed of two to four layers of cells throughout, 88–100 μm in thickness. In transverse section medullary cells, 80–157 μm in height, 15–25 μm in width, are overlain by a single cortical cell, 25–50 μm in height, 15–25 μm in width, and then in longitudinal section, two to three cortical cells over lay each medullary cell. Tetrasporangia are spherical, scattered over the both sides of thallus except the margins, 80–100 *μ* m in height by 85–95 *μ* m in diameter, with one basal stalk cell projecting out from the thallus surface, without forming a sorus, indusia and paraphyses absent.

#### Holotype

The holotype is deposited at Department of Biology, National Changhua University of Education, Changhua (NCUE-CAF91072101) (Figure 2).

#### Type locality

Chuan-Fan-Shih, Southern Taiwan (21˚56′01″N, 120˚49′21″E).

#### Etymology

“formosana” refers to Taiwan, where the alga was collected.

#### Distribution

Known only from southern Taiwan (Figure 1).

#### Habitat and phenology

Absence of perennial stipes indicates that this species may be annual. Plants were found all year round, mainly at 2–5 m depth, where they were abundant on coral reefs or on reef rocks.

#### Specimens examined and localities

**Pingtung County, southern Taiwan:** **Chu-Shui-Kou**, 5–7 m, coll. C-S Lin, CAF91041401, 14 April 2002; **Chuan-Fan-Shih**, 1–4 m, coll. C-C Peng, 840013(NTU), 25 Oct.1995; coll. W-L Wang, CAF85053101, 31 Mar.1996; coll. C-S Lin, CAF90030301, sporophyte, 03 Mar.2001; coll. C-S Lin, CAF90050501, 05 May 2001; coll. S-M Lin, CAF90102601, 26 Oct. 2001; coll. S-M Lin, CAF90112801, sporophyte, 28 Nov. 2001; coll. C-S Lin, CAF91011301, sporophyte, 13 Jan. 2002; coll. C-S Lin, CAF91020601, 06 Feb. 2002; coll. C-S Lin, CAF91030101, 01 Mar. 2002; coll. S-M Lin, CAF91031401, 14 Mar. 2002; coll. C-S Lin, CAF91041301, 13 April 2002; coll. C-S Lin, CAF91061501, 15 June 2002; coll. C-S Lin, CAF91072101, sporophyte, 21 July 2002; coll. C-S Lin (Holotype), CAF91100201, 02 Oct. 2002; coll. S-L Lau, CAF91103001, sporophyte, 30 Oct. 2002; **Hsiao-Wan**, 1–3 m, coll. S-M Lin, CAF82063001, 30 June 1993; coll. W-L Wang, CAF86042601, 26.iv.1997; coll. C-S Lin, CAF91051101, 11.v.2002; coll. C-S Lin, CAF91072001, sporophyte, 20 July 2002; **Hsiang-Chiao-Wan**, 1–3 m coll. S-M Lin, CAF91032901, 29 May 2002; **Feng-Chui-Sha**, 1–5 m, coll. C-S Lin, CAF91051102, 11 May 2002; **Chiu-Peng**, 2–3 m, coll. G-L Lin, CAF82071101, 11 July 1993; coll. G-L Lin, CAF82102901, 29 Oct.1993.

#### Habitat and anatomical structures

Thalli are yellow or dark brown in color, composed of upright blades (Figures 2 and 3), and which basal portions are creeping with a conspicuously rhizoid holdfast. They are 5–23 cm in height and (1-)3-7(-10) cm in width (Figures 2 and 3). Thallii are fan-shaped when young and splitting into numerous bladelets when old. The surfaces of thallus are covered with hyaline hairs that are arranged in interrupted concentric bands (Figure 4), and with the blanketing brown rhizoidal filament at the base (Figure 5). Thallus growth is by a row of marginal meristem cells, which are dark in color (Figure 6). The apical cell is 120–240 *μ* m in length and 70–78 *μ* m in width (Figure 12). The blades are polystromatic, two or four cell layers, with 88–100 *μ* m in thickness. Cortical cells are 25–50 μm in height and 15–25 *μ* m in width. Those cells occurred on either side of two-cell layers of medullary cells, which measure 80–157 *μ* m in height and 15–25 *μ* m in width (Figures 7 and 8). In longitudinal section of thallus, two or three cortical cells overlay a single medullary cell (Figure 8), whereas a single cortical cell overlays each medullary cell in transverse section (Figures 11 and 13).

#### Reproductive structures

Sporangia are scattered over the surface on both sides of the blade (Figures 9 and 10). Tetrasporangia are roughly spherical and projected above the surface of the thallus, 80–100 *μ* m in height and 85–95 *μ* m in diameter, with a basal stalk cell which measured 12–26 *μ* m in height by 17–25 *μ* m in diameter, and lacked indusium and paraphyses (Figures 11 and 13). Gametophytes were not observed.

### Characteristics of gene sequences

**Figure 14:**
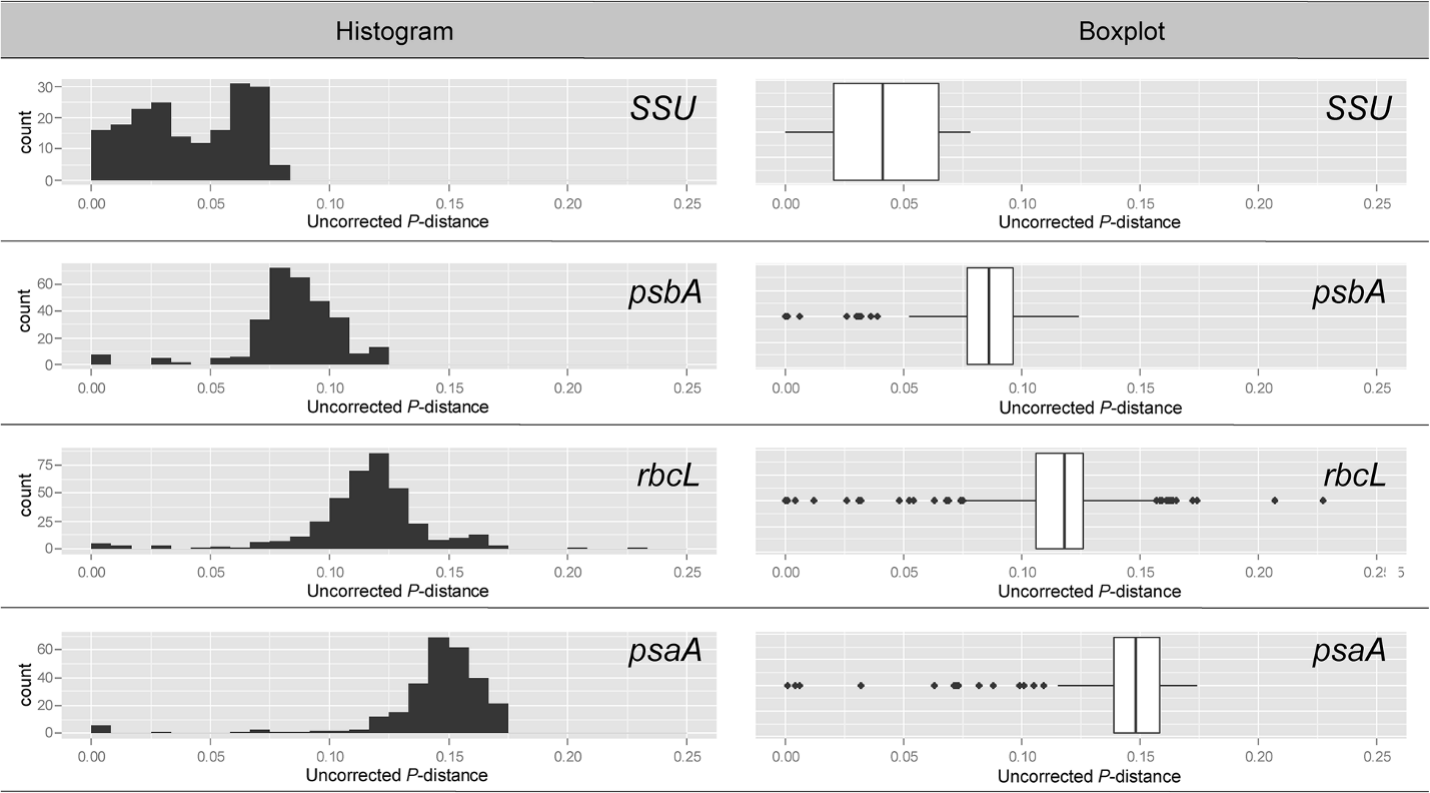
**Histograms and boxplots showing the comparisons of pairwise divergence (uncorrected** ***P*** **-distance) among four different genes.** Hard bars in the boxplots indicate median.

The *SSU* sequences determined and aligned in this study were 1,814 nucleotides long. The 20 aligned *SSU* sequences had 106 (5.8%) variable bases and 176 (9.7%) parsimoniously informative sites and 49.4% G+C contents. Transitions occurred more than transversions (Ts/Tv=1.16). The average of uncorrected pairwise distances (*p*-distances) was 0.059 from the aligned data set (Figure 14). The uncorrected pair wise distance (*p*-distances) between *Zonaria* species and *Homoeostrichus* species ranged from 0.057 to 0.077, and between *Exallosorus* species and *Homoeostrichus* species from 0.009 to 0.014. We could find 5 nucleotide base pairs differences in the aligned 1,723 nucleotide base pairs sequences between *H. formosana* sp. nov. and *H. flabellatus* from Japan (~0.3%), and 11 nucleotide base pairs differences between *H. formosana* sp. nov. and *H. sinclairii.*

We determined and aligned 1,351 nucleotides long *rbcL* sequences in this study. The 28 aligned *rbcL* sequences had 82 (6.07%) variable bases and 420 (31.08%) parsimoniously informative sites. The G+C content was 38.2% in the aligned sequence data set. Transitions were almost less than transversions (Ts/Tv=0.89). The average of *p*-distances was 0.122 from the aligned data set (Figure 14). The “*p*-distance” between *Zonaria* species and *Homoeostrichus* species ranged from 0.118 to 0.125, and between *Exallosorus* species and *Homoeostrichus* species from 0.098 to 0.119. Sixteen nucleotide differences were found between *H. formosana* sp. nov. and *H. flabellatus* in 1,305 nucleotide base aligned sequences (~1.2%).

The *psaA* sequences determined and aligned in this study were 1,395 nucleotides long. The aligned 24 *psaA* sequences had 87 (4.49%) variable bases and 496 (35.55%) parsimoniously informative sites and had 35.1% G+C content, and ratio of 0.82 transitions to transversions (Ts/Tv). The “*p*-distances” was 0.154 from the aligned *psaA* sequences data set (Figure 14). The “*p*-distances” between *Zonaria* species and *Homoeostrichus* species ranged from 0.143 to 0.152, and between *Exallosorus* species and *Homoeostrichus* species from 0.132 to 0.137. We found 182 nucleotide differences between *H. formosana* sp. nov. and *H. sinclairii* in aligned sequences of 1,394 base pairs.

The total 845 base pairs of *psbA* sequences were determined and aligned in this study. The aligned 25 *psbA* sequences had 45 (5.33%) variable bases and 213 (25.21%) parsimoniously informative sites with 37.8% G+C content. Transitions occurred more frequently than transversions (Ts/Tv=1.22) and *p*-distance ranged from 0.030 to 0.134 with average of 0.089 in aligned *psbA* sequences data set (Figure 14). The “*p*-distances” between *Zonaria* species and *Homoeostrichus* species ranged from 0.072 to 0.102, and between *Exallosorus* species and *Homoeostrichus* species from 0.084 to 0.098.

Overall, the sequence divergence is smallest in *SSU*, followed by *psbA* (Figure 14). In contrast, the sequence divergence is much larger in *rbc* L and *psaA* (Figure 14). Our observations suggest that *SSU* is more suitable to resolve the phylogenetic relationship of higher taxonomic level and other plastid genes used in this study are more suitable to tackle the phylogenetic relationship for the lower taxonomic level.

### The phylogeny based on gene sequences

**Figure 15:**
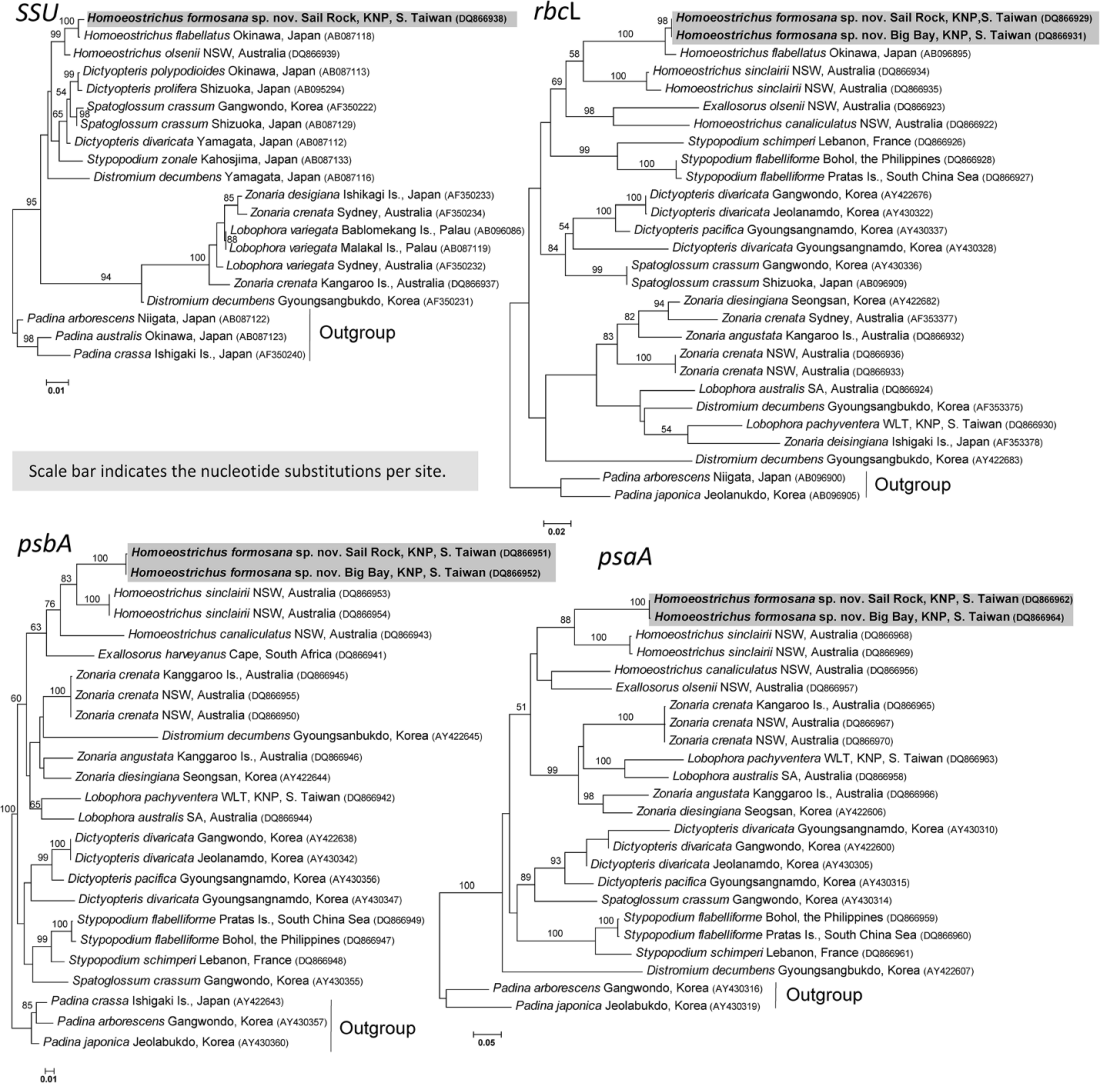
**Maximum likelihood tree for** ***Homoeostrichus formosana*** **sp. nov. and related taxa estimated from the** ***SSU*** **rRNA gene, the** ***rbc*** **L gene, the** ***psaA*** **gene, and the** ***psbA*** **gene.** The statistical support based on 500 bootstrapping replicates of maximum-likelihood analysis are shown on the branches.

The phylogenetic tree based on *SSU* sequences showed that genera of tribe Zonarieae made four clades with no phylogenetic resolution among them in the ML analyses (Figure 15). The clade of *Homoeostrichus* and *Exallosorus* species is separated from that of *Zonaria* and *Lobophora* species. Three *Homoeostrichus* species made a subclade distinguished from *Exallosorus* species except for *H. canaliculatus*. Especially *H. formosana* sp. nov. made a clade with *H. flabellatus* with very low supporting value in three analyses.

The topology of phylogenetic tree based on *rbcL* sequences also show that four clades are distinguished (Figure 15). The clade comprising *Homeostrichus* and *Exallosorus* species figured out as basal sister group in this phylogeny although the results showed pale phylogenetic resolution. *Homoeostrichus formosana* sp. nov. made a clade with *H. flabellatus* with very high supporting value, and a sister group of *H. sinclairii* with low supporting value in three analyses. *Exallosorum olsenii* also made a sister group to three *Homoeostrichus* species clade and closely related to *H. cnaliculatus*. The clade of *Zonaria* and *Lobophora* species made a concrete clade with high supporting value distinguished from others.

The aligned *psaA* gene sequences data set made the phylogenetic tree with five clades, which have a basal clade of *Stypopodium* species although with pale phylogenetic resolution (Figure 15). As in the former trees, the clade of *Homeostrichus* and *Exallosorus* species is distinguished as basal sister group in this phylogeny although having pale phylogenetic resolution. *Homoeostrichus formosana* sp. nov. made a clade with *H. flabellatus* with very high supporting value, with a sister group of *H. sinclairii* with low supporting value in three analyses. *Exallosorum olsenii* also made a sister calde with three *Homoeostrichus* species and closely related to *H. cnaliculatus* as in the phylogeny of *rbcL*. The clade of *Zonaria* and *Lobophora* species made a concrete clade with high supporting value distinguished from *Homoeostrichus* and *Exallosorus* species.

The phylogenic tree based on *psbA* gene also show that *H. formosana* sp. nov. is involved in a clade with *H. sinclairii* and *H. canaliculatus*. This phylogenic tree is composed of five clades with very pale phylogenic resolution (Figure 15). *Exallosorus* species are closely related to *Homoeostrichus* species as in the other phylogenic trees.

## Discussion

The taxonomy of the Dictyotales is largely based on the comparison of vegetative and reproductive growth and organization (Phillips 1997). *Homoeostrichus formosana* sp. nov*.* is mainly characterized by blades composing of two to four layers of cells, single tetrasporangia scattered over both thallus surfaces, sporangia borne on a stalk cell, and lacking indusium and paraphyses. In the erect to recumbent fan-like fronds of *Lobophora*, unusual large medullary cell and indusiate sporangial sorus, and *Padina*, rolling margin and concentric arrangement of reproductive structures, which both are conspicuously differed from *Homoeostrichus*, whatever the habit, texture, anatomical and reproductive structures. *Homoeostrichus* has been very easily confused with *Exallosorus* and *Zonaria*, based on the vegetative and reproductive characters used for separating among them summarized in Table 2. The phylogenic trees based on *SSU*, *psaA, psbA,* and *rbcL* gene sequences supported that *Homoeostrichus* species are closely related to *Exallosorus* species but clearly separated from each others in addition to *Zonaria* species.

**Table 2:** **Comparative features of the genera** ***Exallosorus*** **,** ***Homoeostrichus*** **, and** ***Zonaria***

	***Exallosorus***	***Homoeostrichus***	***Zonaria***
No. of cell layers	4–6	2–4–7	4–12
No. of cortex / medulla in transverse section	1	1	2
Sporangia	Sorus	Single, Sorus	Sorus
No. of stalk cells	1	1 or more	0
No. of spores	4	4	8
Indusium	+	-	+
Paraphyses	+ or -	+ or -	+
Color	Brown	Brown	White
Structure	Spherical cells	Spherical cells	Elliptic cells in upper, spherical cells in middle and base
Distribution	Australia and South Africa	Australia and Taiwan	widespread
References	b, c	a, b, c, this study	a, b, c

,^1987^; b: Phillips et al., 1994; c: Phillips, 1997; +: Present; -: Absent.

The genus *Exallosorus* is separated from *Zonaria* and *Homoeostrichus* in having regularly arranged cells in transverse section, densely placed basally stalked sporangia within sori that possess brown paraphyses and indusium (Phillips 1997) (Table 2). Sporangia of *Zonaria* lacked basal stalk cells, are surrounded by whitish paraphyses (except in *Z. angustata*) in the indusiate sori, and released eight spores (Womersley 1987; Phillips et al. 1994; Phillips 1997) (Table 2). Sporangia of *Homoeostrichus* are distributed among brown paraphyses in non-indusiate sori, and released four spores (Womersley 1987; Phillips et al. 1994; Phillips 1997) (Table 2). In this study, we also found the sporangia in *H*. *formosana* sp. nov. are singly scattered over the surfaces of the thallus without forming a sorus and lacking indusium and paraphyses (Table 3). Classifying the genera of tribe Zonariae based on these morphological and anatomical characteristics is basically agreed to five clades in phylogenic analyses based on gene sequences.

**Table 3:** **Comparisons of vegetative and sporangial structures among the species of** ***Exallosorus*** **,** ***Homoeostrichus*** **and** ***Zonaria***

Characters	***H. formosana***	***H. canaliculatus***	***H. sinclairii***	***E. harveyanus***	***E. olsenii***	***Zonaria*** spp.
Thallus thickness	88–100 μm	150–200 μm	105–180 μm	120–170 μm	90–115 μm	66–300 μm
No. of cell layers	2–4	6–7	4–7	6	4–6	
Cortical /Medullary cells	1	1(2)	1(2)	1(2)	1(2)	2(1)
Sporangia	Tetra-, Single	unknown	Tetra-, Sorus	Tetra-, Sorus	Tetra-, Sorus	Octo-, Sorus
Indusium	-	x	-	+	+	+
Paraphyses	-	x	Brown, in sorus	-	Brown, near sorus	Whitish, in sorus
Stalk cell	1	x	Multicellular	1	1	-
Oogonia	unknown	unknown	Sorus	unknown	Sorus	Sorus
Indusium	x	x	-	x	+	+
Paraphyses	x	x	Brown, among oogonia	x	Brown, near sorus	-
Stalk cell	x	x	-	x	1	1
Antheridial sorus	unknown	unknown	sorus	unknown	sorus	sorus
Sorus border	x	x	Brown paraphyses and sterile filaments	x	Cortical cells	Slightly elongate sterile cells
Stalk cell	x	x	1	x	1	1
References	f	b	b, c, d, e	a, e	b, d, e	b, c, d, e

,^1964^; b: Womersley, 1987; c: Phillips et al., 1994; d: Phillips and Clayton, 1997; e: Phillips; 1997; f: This study. +: Present, -: Absent, x: not found.

*Homoeostrichus formosana* sp. nov. is superficially similar to *Zonaria diesingiana* found from Taiwan in external morphology. However, *H. formosana* sp. nov. can be distinguished vegetatively and reproductively from *Z. diesingiana*, especially it makes four cell layers. The thallus of *Z. diesingiana* is composed of 4–8 layers of cells, in which the one medullary cell is flanked by 2 cortical cells in transverse section, the octosporangia are borne on no stalk cell, and white paraphyses are present in indusiate sori. However, the tetrasporangium of *H. formosana* sp. nov. is borne on a basal stalk cell and lacks paraphyses and indusium.

*Homoeostrichus formosana* sp. nov. was previously misidentified as *E. harveyanus* (as *Z. harveyana*, *H. multifidus*) in Taiwan (Yamada 1925; Okamura 1936; Shen and Fan 1950; Chiang 1960; Lewis and Norris 1987). The thallus of *E. harveyanus* is composed of 6 layers of cells, which measured 120–170 *μ* m in thickness, and the sporangia are formed in a dark brown band of an indusiate sorus, whereas the sporangia in *H*. *formosana* are singly scattered over the surfaces of the thallus without forming a sorus (see Table 3). Although Yamada (1925) and Okamura (1936) had documented the thallus of “*Homoeostrichus multifidus*” (as *H. formosana* sp. nov. in this study) as being composed of four layers of cells, they did not observe reproductive structures, moreover, it is now known that *E. harveyanus* (as *H. multifidus*) is only distributed in southern Africa, the type locality (Silva et al. 1996; Phillips 1997). All molecular data also supported that *H. formonosa* sp. nov. is clearly distinguished from *E. harveyanus* in the *psbA* sequences molecular analyses in this study. Another *Exallosorus* species, *E. olsenii*, comprised of six cell layers, has sporangia assembled in indusiate sori that are connected with hairs and paraphyses, and with the reproductive structures only occurring on one thallus surface (Womersley 1987, as *H. olsenii*; Phillips et al. 1994; Phillips 1997), which is not agreed with *H. formosana* sp. nov. (Table 3).

*Homoeostrichus formosana* sp. nov can possibly be confused with other species of *Homoeostrichus*: *H. canaliculatus* (Womersley and *H. sinclairii*^1987^; Phillips 1997). However, *H. formosana* sp. nov. can be distinguished from the other species of *Homoeostrichus* by its 2–4 layers of cells thallus and sporangial stalk cells opposed to a 6–7 cell layer thallus and by multicellular stalk cells which are found in *Homoeostrichus* (see Table 3). The phylogentic tree especially based on *psbA* gene sequences showed that *H. canaliculatus* is distinguished from other *Homeostrichus* species and from *Exallosorus* species. Moreover, *H. flabellatus* Okamura, another Dictyotaceae species from Taiwan, might also be confused with *H. formosana* sp. nov. (Taniguti 1976; Lewis and Norris 1987; Wang and Chiang 2001). Okamura (1936) reported the thallus of *H. flabellatus* was composed of three layers of cells but he did not observe reproductive structures. Womersley (1987) speculated that Japanese *H. flabellatus* did not belong to the genus *Homoeostrichus*, and Papenfuss (1944) transferred *H. flabellatus* to *Zonaria flabellata* (Okamura) C. However, this combination is not recognized by some phycologists (see Phillips 1997; Phillips and Nelson 1998). The molecular characteristics of SSU show Japanese *H. flabellatus* is more related to *H. formosana* sp. nov. in this study. These show that the status of this taxon should be required further study especially examining voucher specimens of *H. flabellatus*. Furthermore, it is noted that an undescribed *Zonaria* sp. was recently reported from Chaojing, Keelung, northern Taiwan by Kitayama and Lin (2012). Though they only showed single photo of the thallus of this alga without any anatomical observations, this alga is highly similar to *H. formasana* in appearance. Considering that *H. flabellatus* (as *Zonaria flabellatus*) in Okinawa is biogeographically close to northern Taiwan (Figure 15), it will be interesting to examine the phylogenetic affinity of this undescribed *Zonaria* sp. from the northern Taiwan to test whether this alga is phylogenetically close to *H. flabellatus* or *H. formasana*.

## Conclusions

We describes a new species, *Homoeostrichus formosana* Wang, Lin, Lee *et* Liu, collected from Taiwan. This species has marginal row of apical cells responsible for thallus growth and the thallus with four layers of cells except the marginal regions. The cortical cell lies upon each medullary cell in transverse section, and two cortical cells upon each medullary cell in longitudinal section. Tetrasporangium is observed for the first time, which is developed from cortical cell with stalk cell and singly scattered over the thallus surface, and has no indusia and paraphyses. The phylogenetic trees based on *SSU*, *psaA, psbA,* and *rbcL* gene sequences supported that *Homoeostrichus* species are closely related to *Exallosorus* species but distinctly different from *Zonaria* species.

## Authors’ original submitted files for images

Below are the links to the authors’ original submitted files for images. Authors’ original file for figure 1 Authors’ original file for figure 2 Authors’ original file for figure 3 Authors’ original file for figure 4 Authors’ original file for figure 5 Authors’ original file for figure 6 Authors’ original file for figure 7 Authors’ original file for figure 8 Authors’ original file for figure 9 Authors’ original file for figure 10 Authors’ original file for figure 11 Authors’ original file for figure 12 Authors’ original file for figure 13 Authors’ original file for figure 14 Authors’ original file for figure 15

## References

[CR1] Agardh CA (1817). Synopsis algarum scandinaviae.

[CR2] Agardh JG (1894). Analecta alglogica. Continuatio I. Lunds universitets Års-skrift andra afdelningen, kongl. Fysiografiska sällskapets I. Lund Handlingar.

[CR3] Allender BM, Kraft GT (1983). The marine algae of lord Howe island (New south Wales): the *Dictyotales* and *Cutleriales* (Phaeophyta). Brunonia.

[CR4] Børgesen F (1926). Marine algae from the canary islands, especially from Teneriffe and Gran Canaria, II: *Phaeophyceae*. Biol Meddelelser.

[CR5] Chiang YM (1960). Marine algae of northern Taiwan (Cyanophyta, Chlorophyta, Phaeophyta). Taiwania.

[CR6] Chun J (1995). Ph. D. Thesis. Computer-assisted classification and identification of actinomycetes.

[CR7] Farrant PA, King RJ (1989). The *Dictyotales* (algae: phaeophyta) of New South Wales. Proc Linnean Soc New South Wales.

[CR8] Gayral P (1966). Dion. Les algues de côte francaises.

[CR9] Hoshina R, Hasegawa K, Tanaka J, Hara Y (2004). Molecular phylogeny of the dictyotaceae (*Phaeophyceae*) with emphasis on their morphology and its taxonomic implication. Jpn J Phycol.

[CR10] Hwang IK, Kim HS, Lee WJ (2004). Confirmation on taxonomic status of *Spatoglossum pacificum* yendo (Dictyotaceae, *Phaeophyceae*) based on morphology and plastid protein coding *rbcL*, *rbcS*, *psaA*, and *psbA* gene sequences. Algae.

[CR11] Hwang IK, Kim HS, Lee WJ (2005). Polymorphism in the brown alga *Dictyota dichotoma* (Dictyotaceae, *Dictyotales*) from Korea. Mar Biol.

[CR12] Kitayama T, Lin S-M (2012). Brown alga from Chaojing, Keelung City, Taiwan. Mem Natl Mus Sci, Tokyo.

[CR13] Lee WJ, Bae KS (2002). Phylogenetic relationship among several genera of Dictyotaceae (*Dictyotales*, *Phaeophyceae*) based on 18S rRNA and *rbcL* gene sequences. Mar Biol.

[CR14] Lewis JE, Norris JN (1987). A history and annotated account of the benthic marine algae of Taiwan. Smithson Contr Mar Sci.

[CR15] Okamura K (1936). Nippon Kaiso-si.

[CR16] Papenfuss GF (1944). Notes on algal nomenclature. II. Miscellaneous species of *Chlorophyceae*, *Phaeophyceae* and Rhaeophyta. Farlowia.

[CR17] Phillips JA (1997). Genus and species concepts in *Zonaria* and *Homoeostrichus* (*Dictyotales*, *Phaeophyceae*), including the description of *Exallosorus* gen. nov. Eur J Phycol.

[CR18] Phillips JA, Clayton MN (1993). Comparative flagellar morphology of spermatozoids of the *Dictyotales* (*Phaeophyceae*). Eur J Phycol.

[CR19] Phillips JA, Clayton MN (1994). Flagellate spores in *Homoeostrichus olsenii* Womersley (*Dictyotales*, *Phaeophyceae*): the largest know motile reproductive cells of marine macroalgae. Phycologia.

[CR20] Phillips JA, Clayton MN (1997). Comparative studies on gametangial distribution and structure in species of *Zonaria* and *Homoeostrichus* (*Dictyotales*, *Phaeophyceae*) from Australia. Eur J Phycol.

[CR21] Phillips JA, Nelson WA (1998). Typification of the Australasian brown alga *Zonaria turneriana* J. Agardh (*Dictyotales*) and description of the endemic New Zealand species, *Zonaria aureomarginata* sp. nov. Bot Mar.

[CR22] Phillips JA, Clayton MN, Harvey AS (1994). Comparative studies on sporangial distribution and structure in species of *Zonaria*, *Lobophora* and *Homoeostrichus* (*Dictyotales*, *Phaeophyceae*) from Australia. Eur J Phycol.

[CR23] Ribera MA, Gómez-Garreta A, Gallardo T, Comez-Garreta M, Gallardo T, Cormaci M, Furnari G, Giaccone G (1992). Check-list of Mediterranean seaweeds. I. Fucophyceae (Warming 1884). Bot Mar.

[CR24] Seagarief SC (1984). A catalogue of south African green, brown and red marine algae. Mem Bot Surv South Africa.

[CR25] Shen YF, Fan KC (1950). Marine algae of Formosa. Taiwania.

[CR26] Silva PC (1952). A review of nomenclatural conservation in the algae from the point of view of the type method. Univ Calif Pub Bot.

[CR27] Silva PC, Menez G, Moe RL (1987). Catalog of benthic marine algae of the Philippines. Smithson Contr Mar Sci.

[CR28] Silva PC, Basson PW, Moe RL (1996). Catalogue of the benthic marine algae of the Indian ocean. Univ California Publ Bot.

[CR29] Simons RH (1964). Notes on the species of *Zonaria* in south Africa. Bothalia.

[CR30] Tamura K, Peterson D, Peterson N, Stecher G, Nei M, Kumar S (2011). MEGA5: molecular evolutionary genetics analysis using maximum likelihood, evolutionary distance, and maximum parsimony methods. Mol Biol Evol.

[CR31] Taniguti M (1976). Phytosociological study of marine algae in Taiwan. Bull Mie Univ.

[CR32] Taylor WR (1960). Marine algae of the eastern tropical and subtropical coasts of the Americas.

[CR33] Wang WL, Chiang YM (2001). The marine macroalgae of Lu Tao (green island), Taiwan. Taiwania.

[CR34] Womersley HBS (1987). The marine benthic flora of southern Australia part II.

[CR35] Yamada Y (1925). Studien über die meeresalgen von der insel Formosa 2. Phaeophyceae Bot Mag (Tokyo).

[CR36] Yoon HS, Hackett JD, Bhattacharya D (2002). A single origin of the peridinin- and fucoxanthin-containing plastids in dinoflagellates through tertiary endosymbiosis. Proc Natl Acad Sci USA.

[CR37] Yoshida T (1998). Marine algae of Japan.

[CR38] Yoshida T, Nakajima Y, Nakata Y (1985). Preliminary checklist of marine benthic algae of Japan-I. *Chlorophyceae* and *Phaeophyceae*. Jap J Phycol.

