# Supplementary figures and images for: Morphological and molecular characteristics of Homoeostrichus formosana sp. nov. (Dictyotaceae, Phaeophyceae) from Taiwan
Source: Bot Stud. 2013 Aug 21;54:13. doi: 10.1186/1999-3110-54-13 (PMC5430593; doi:10.1186/1999-3110-54-13)

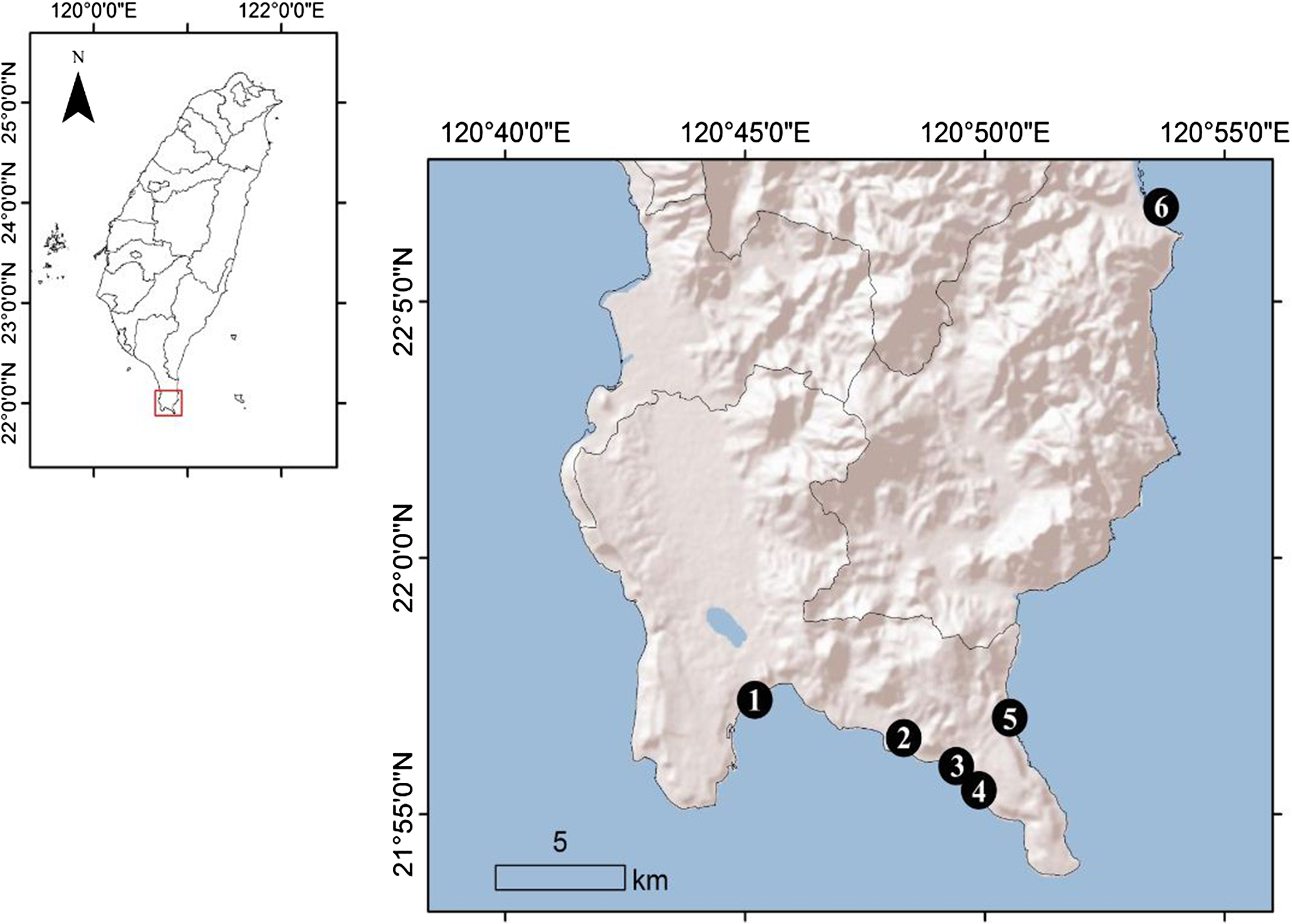

Supplement: Supplementary file 1 — Authors’ original file for figure 1 [file 40529_2012_12_MOESM1_ESM.tif]

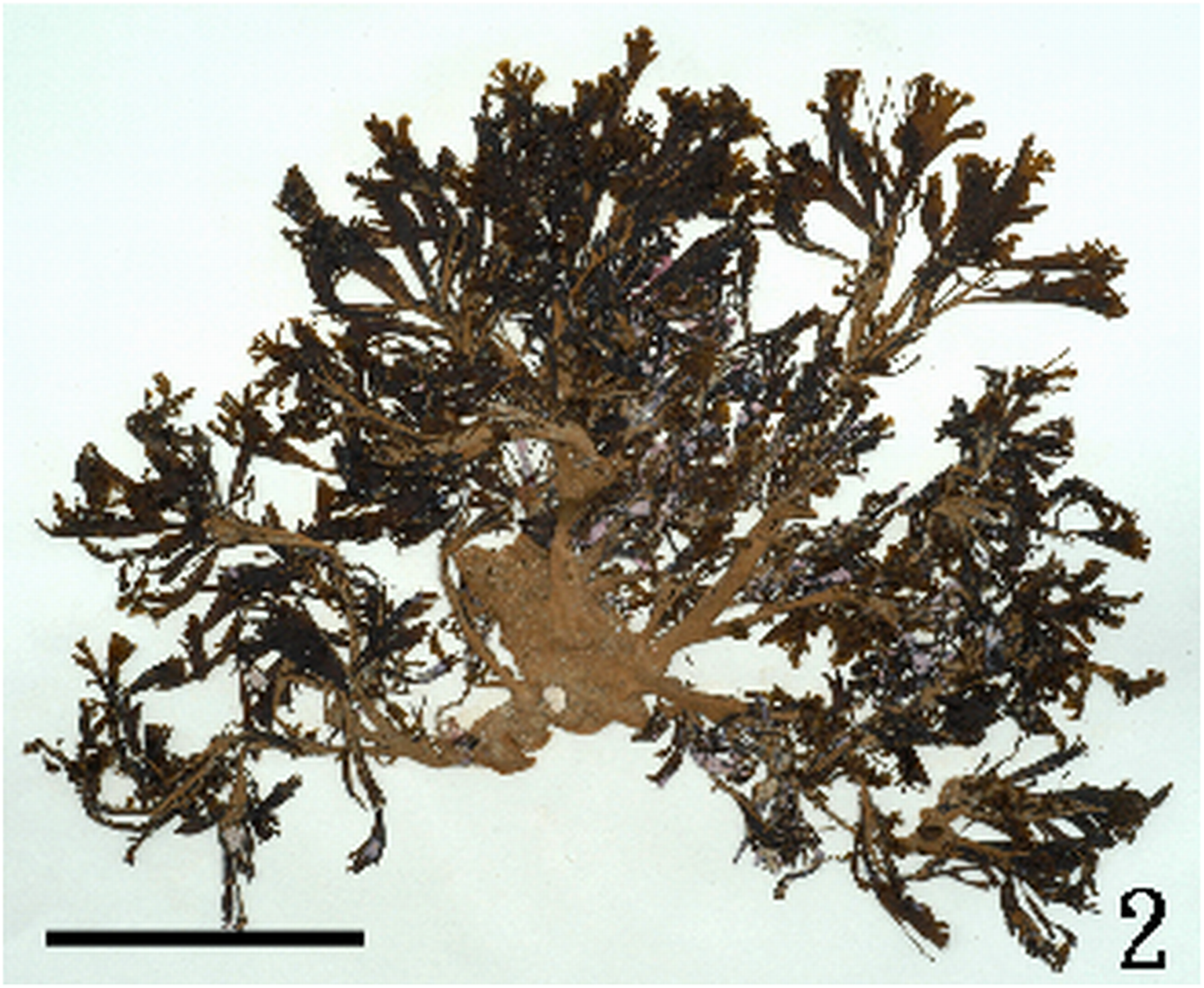

Supplement: Supplementary file 2 — Authors’ original file for figure 2 [file 40529_2012_12_MOESM2_ESM.tif]

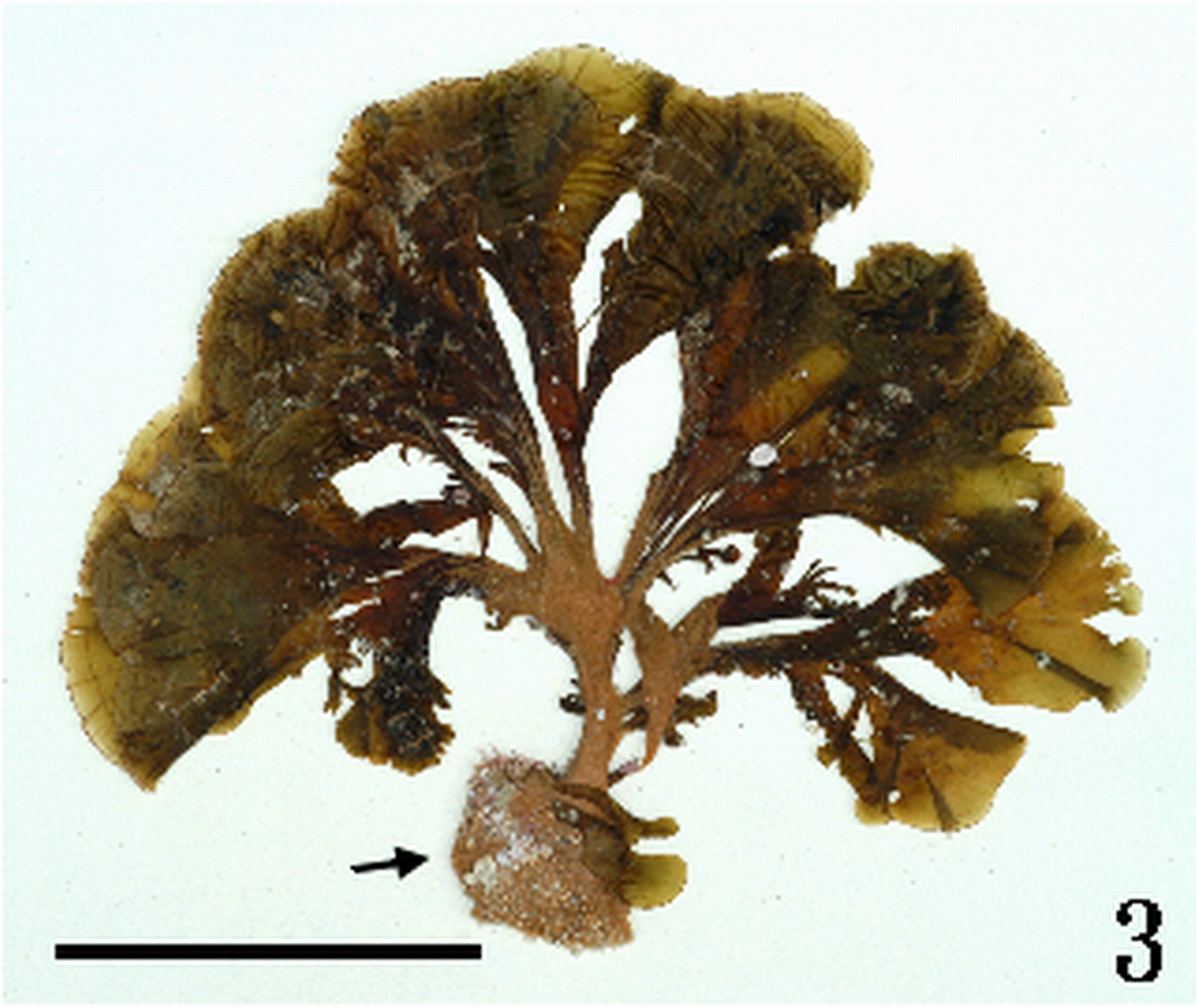

Supplement: Supplementary file 3 — Authors’ original file for figure 3 [file 40529_2012_12_MOESM3_ESM.tif]

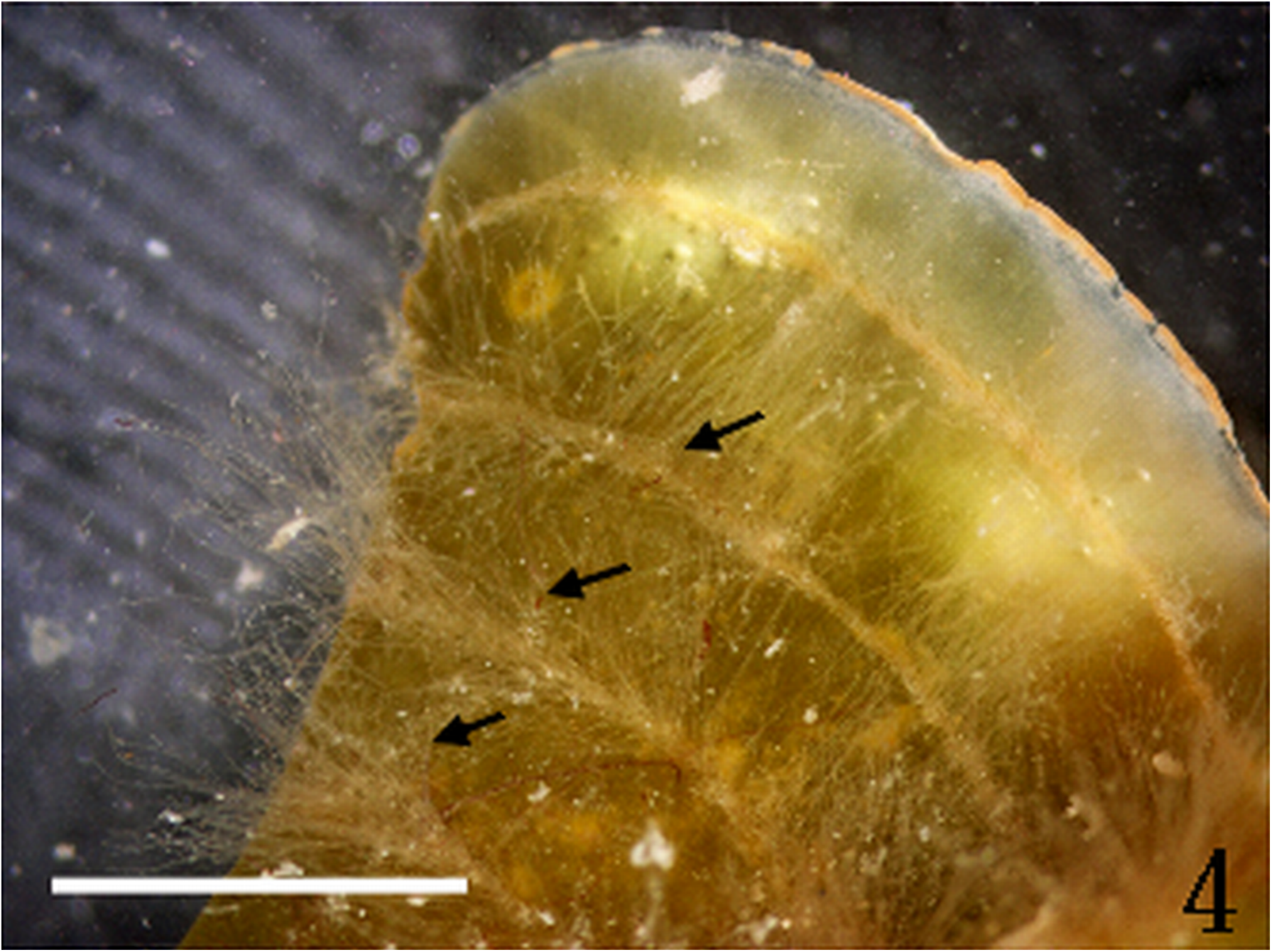

Supplement: Supplementary file 4 — Authors’ original file for figure 4 [file 40529_2012_12_MOESM4_ESM.tif]

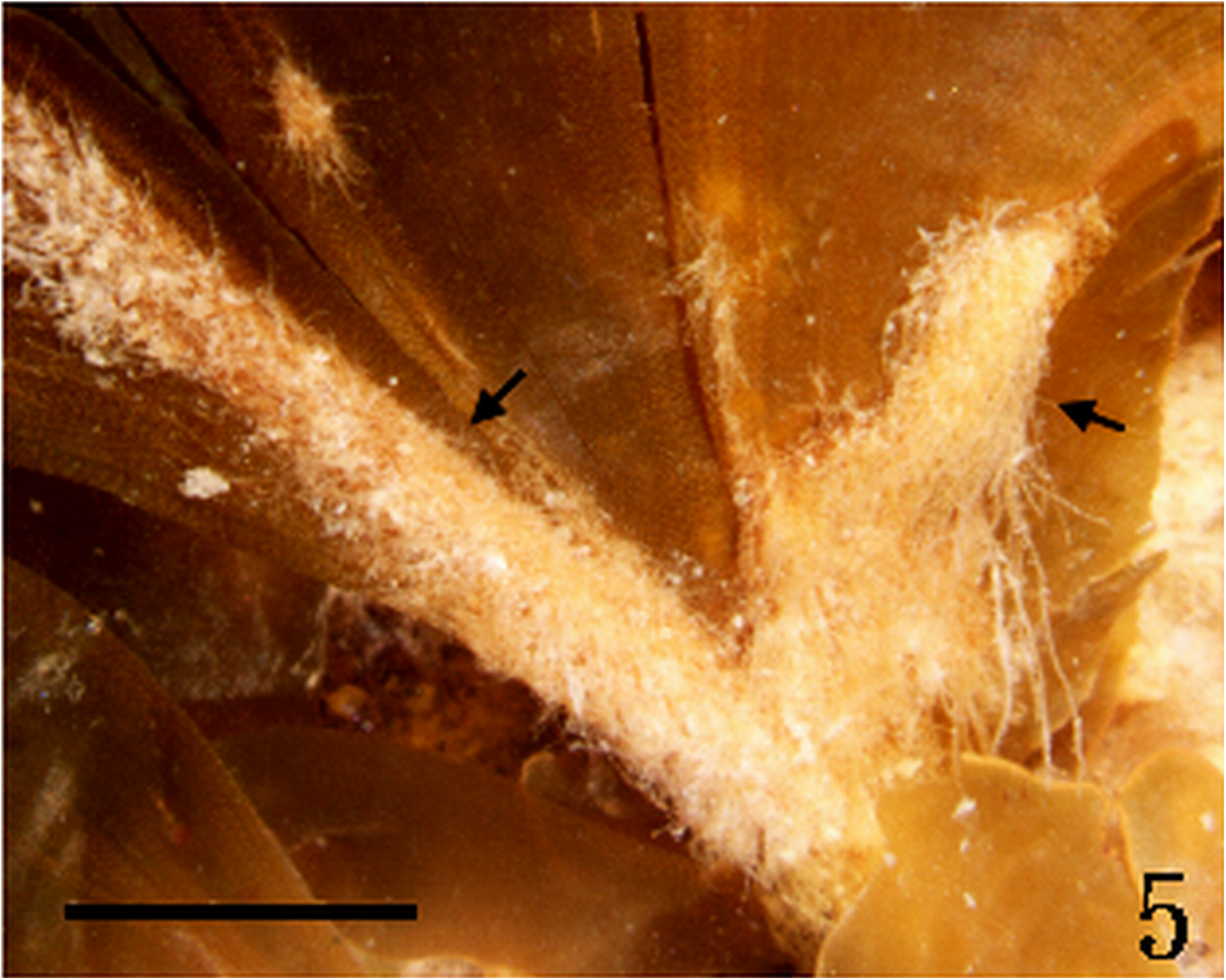

Supplement: Supplementary file 5 — Authors’ original file for figure 5 [file 40529_2012_12_MOESM5_ESM.tif]

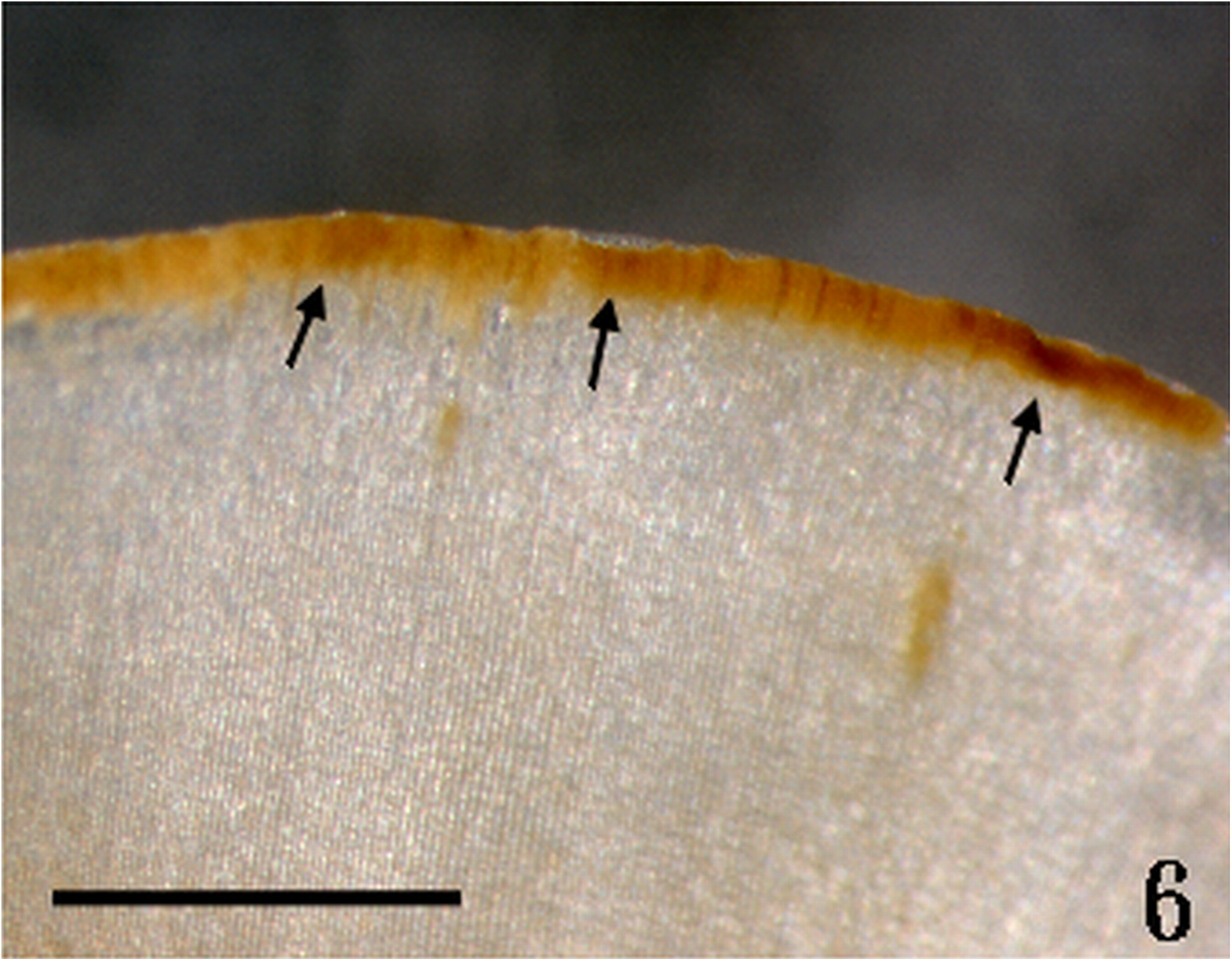

Supplement: Supplementary file 6 — Authors’ original file for figure 6 [file 40529_2012_12_MOESM6_ESM.tif]

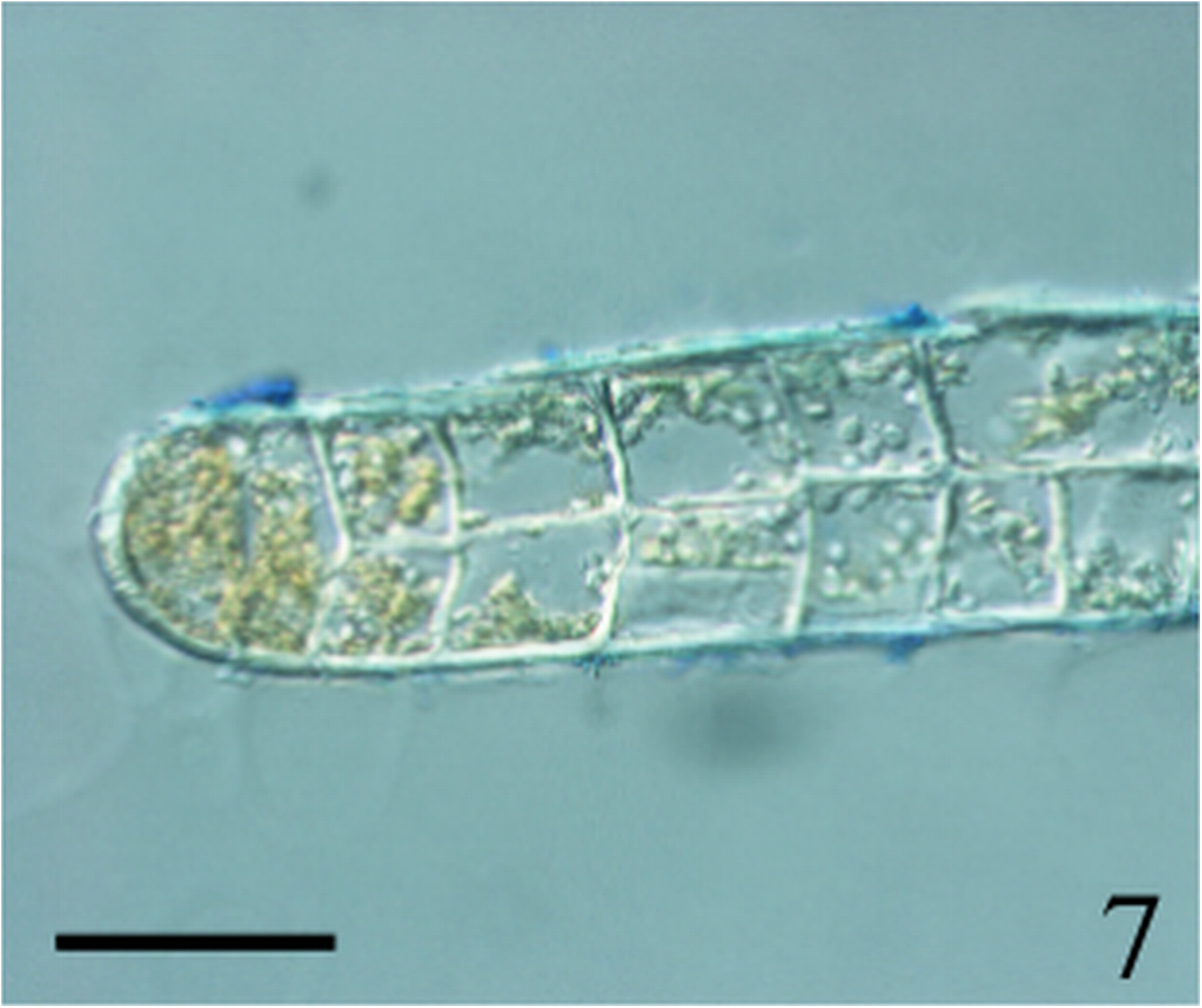

Supplement: Supplementary file 7 — Authors’ original file for figure 7 [file 40529_2012_12_MOESM7_ESM.tif]

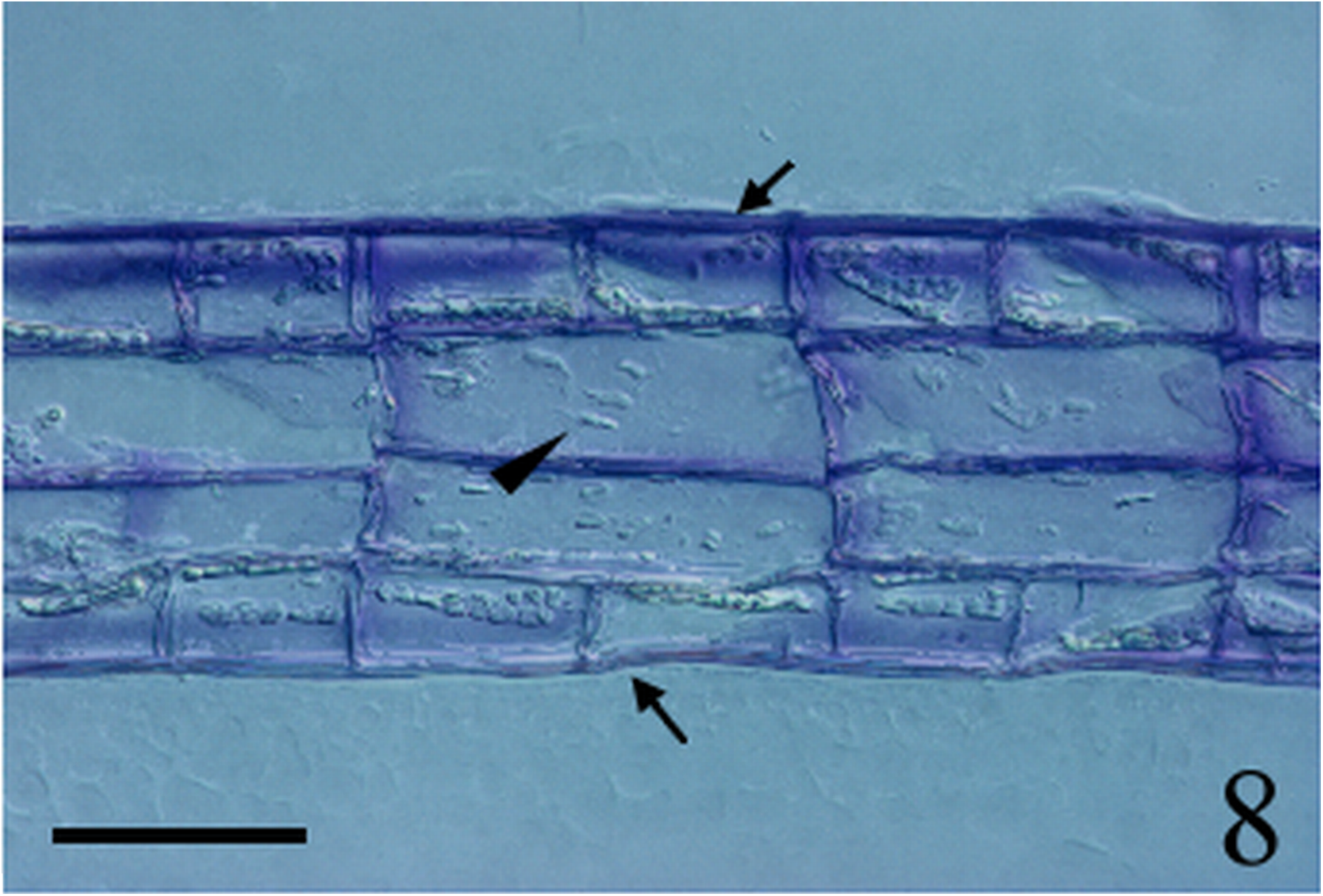

Supplement: Supplementary file 8 — Authors’ original file for figure 8 [file 40529_2012_12_MOESM8_ESM.tif]

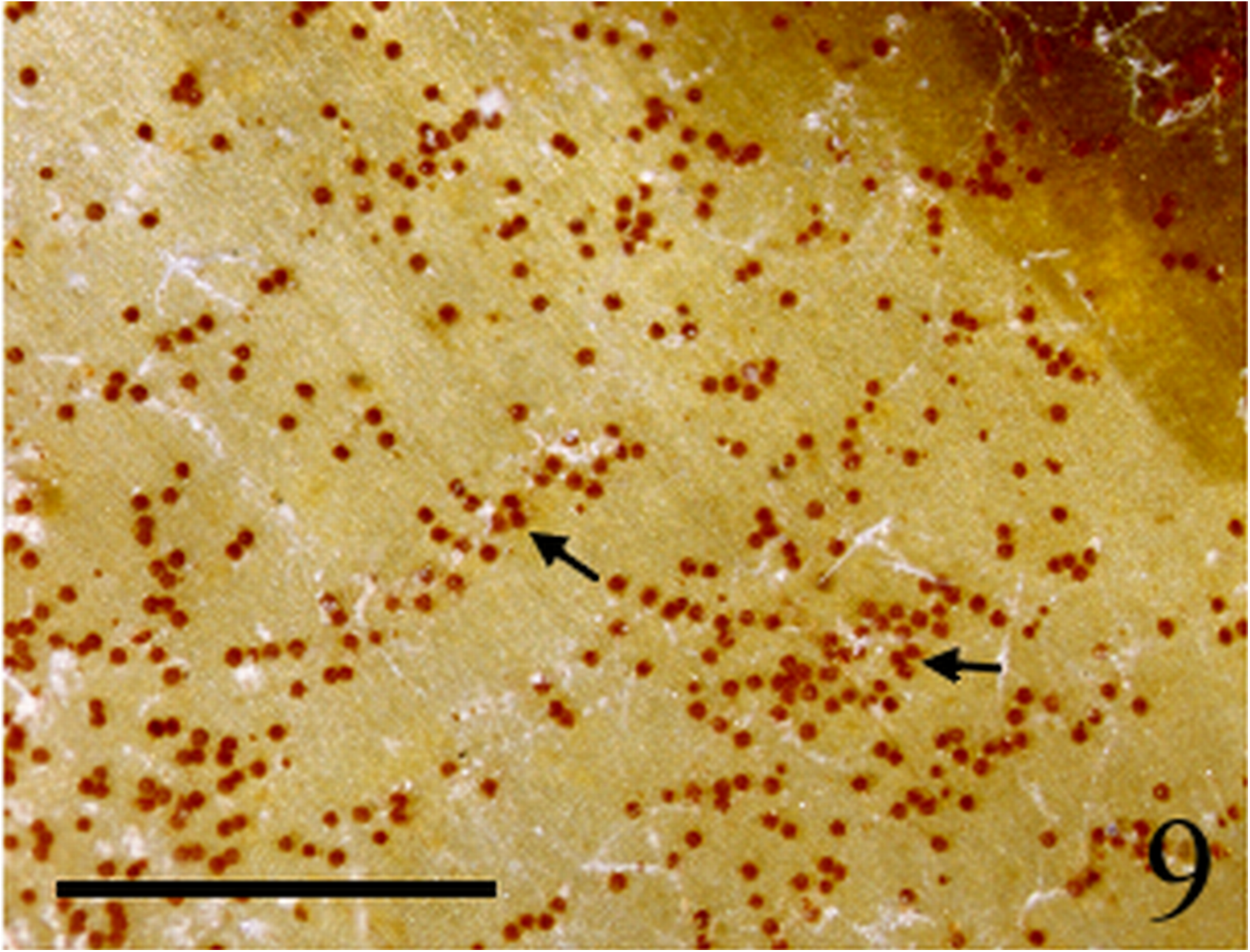

Supplement: Supplementary file 9 — Authors’ original file for figure 9 [file 40529_2012_12_MOESM9_ESM.tif]

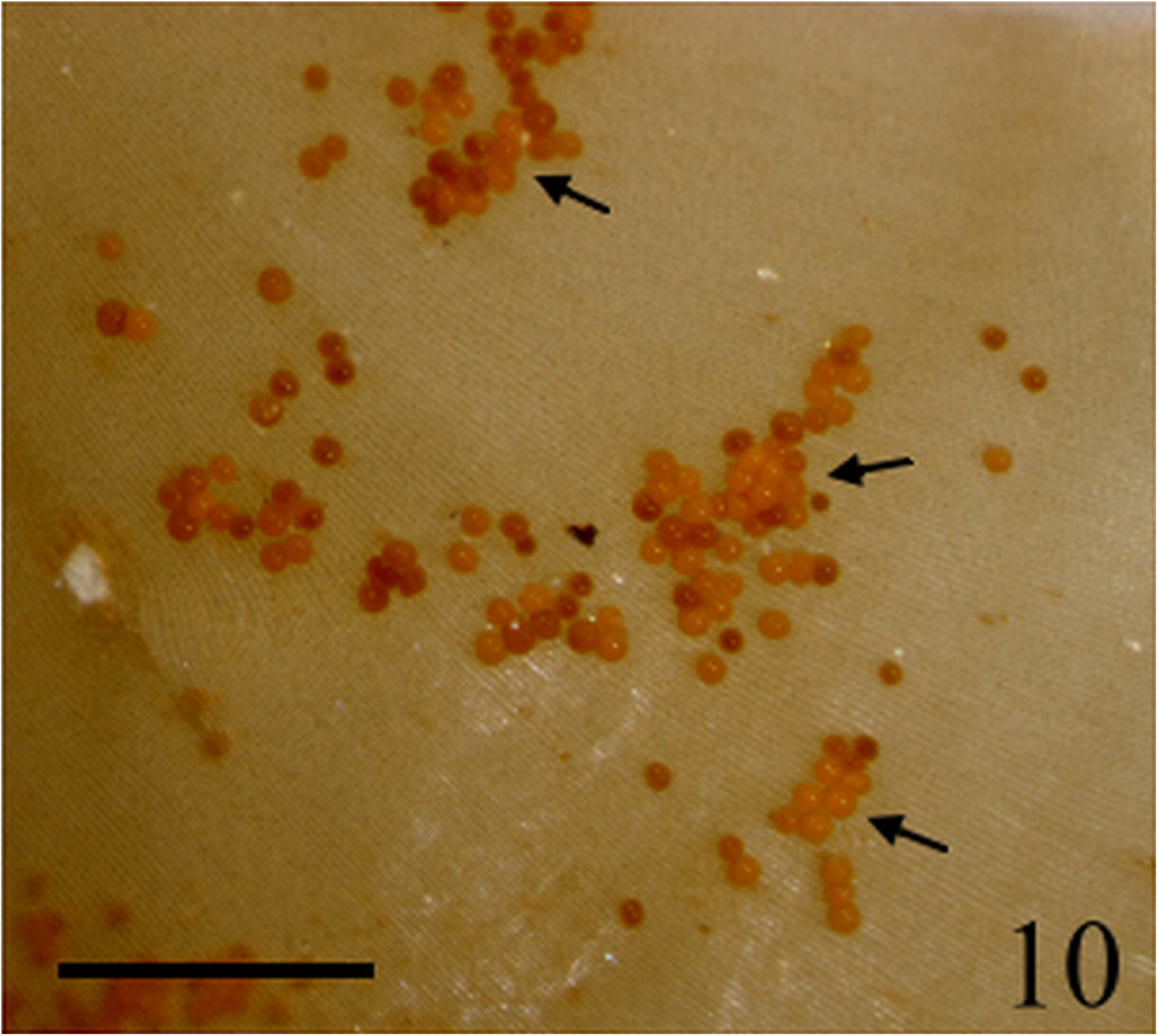

Supplement: Supplementary file 10 — Authors’ original file for figure 10 [file 40529_2012_12_MOESM10_ESM.tif]

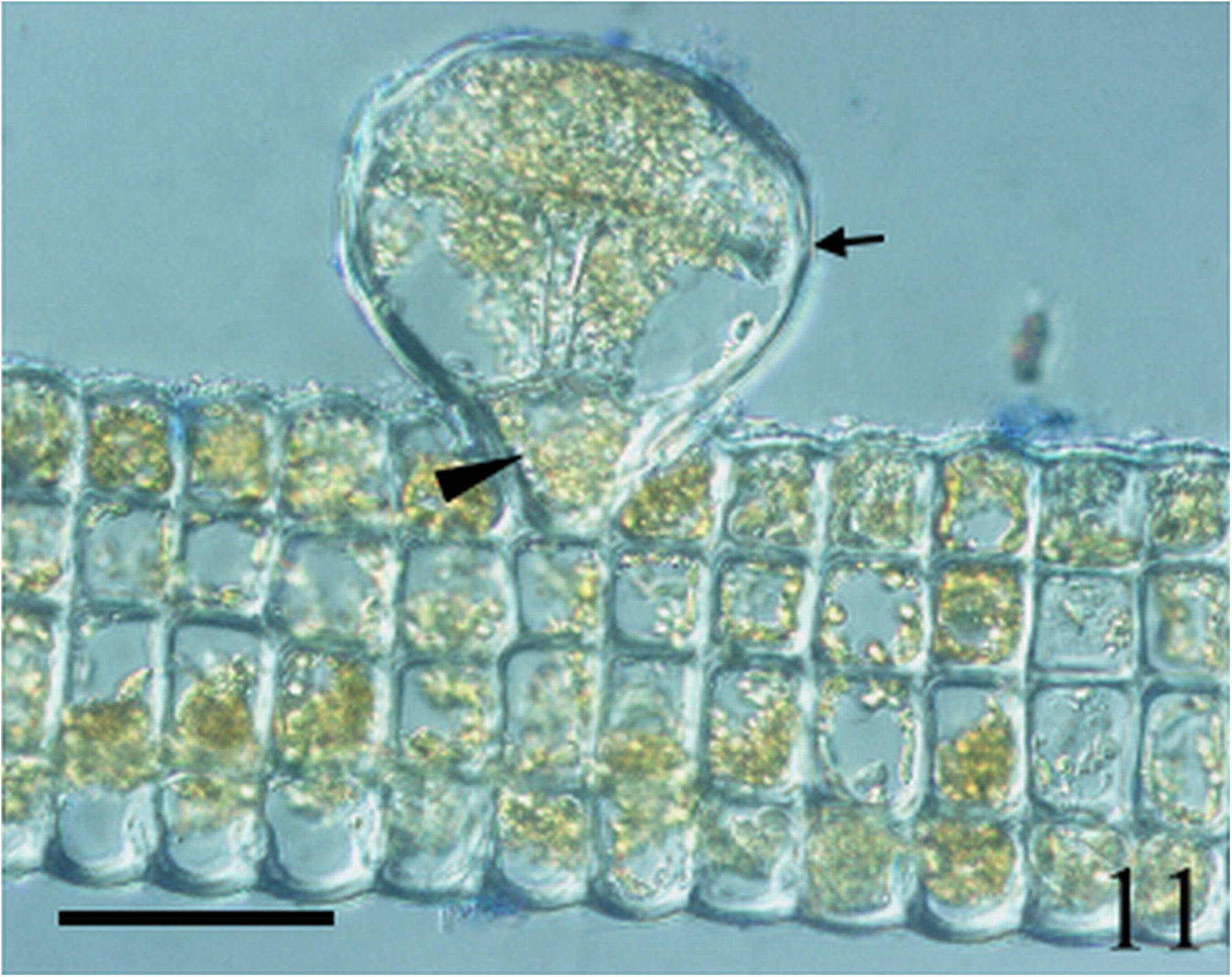

Supplement: Supplementary file 11 — Authors’ original file for figure 11 [file 40529_2012_12_MOESM11_ESM.tif]

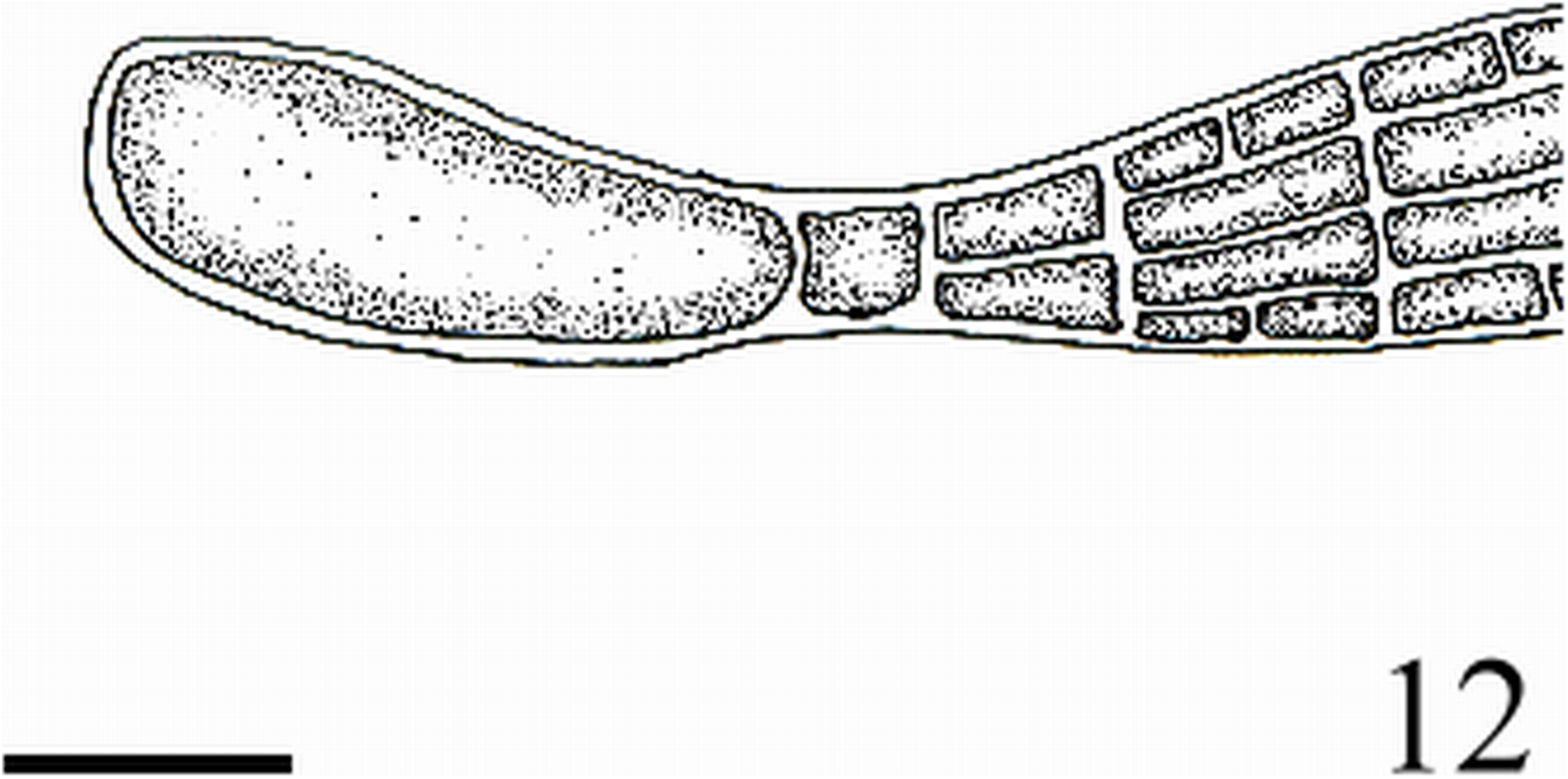

Supplement: Supplementary file 12 — Authors’ original file for figure 12 [file 40529_2012_12_MOESM12_ESM.tif]

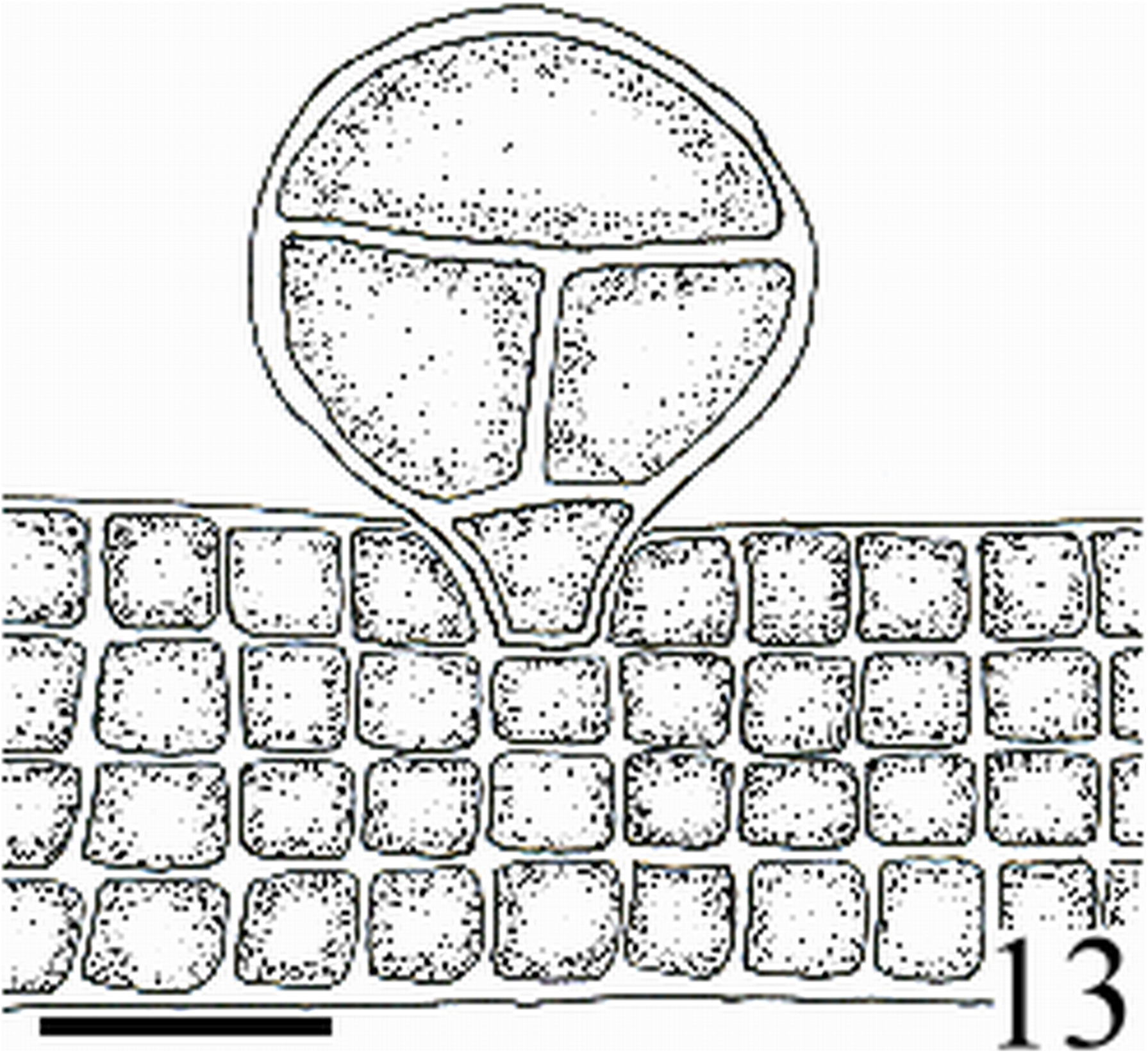

Supplement: Supplementary file 13 — Authors’ original file for figure 13 [file 40529_2012_12_MOESM13_ESM.tif]

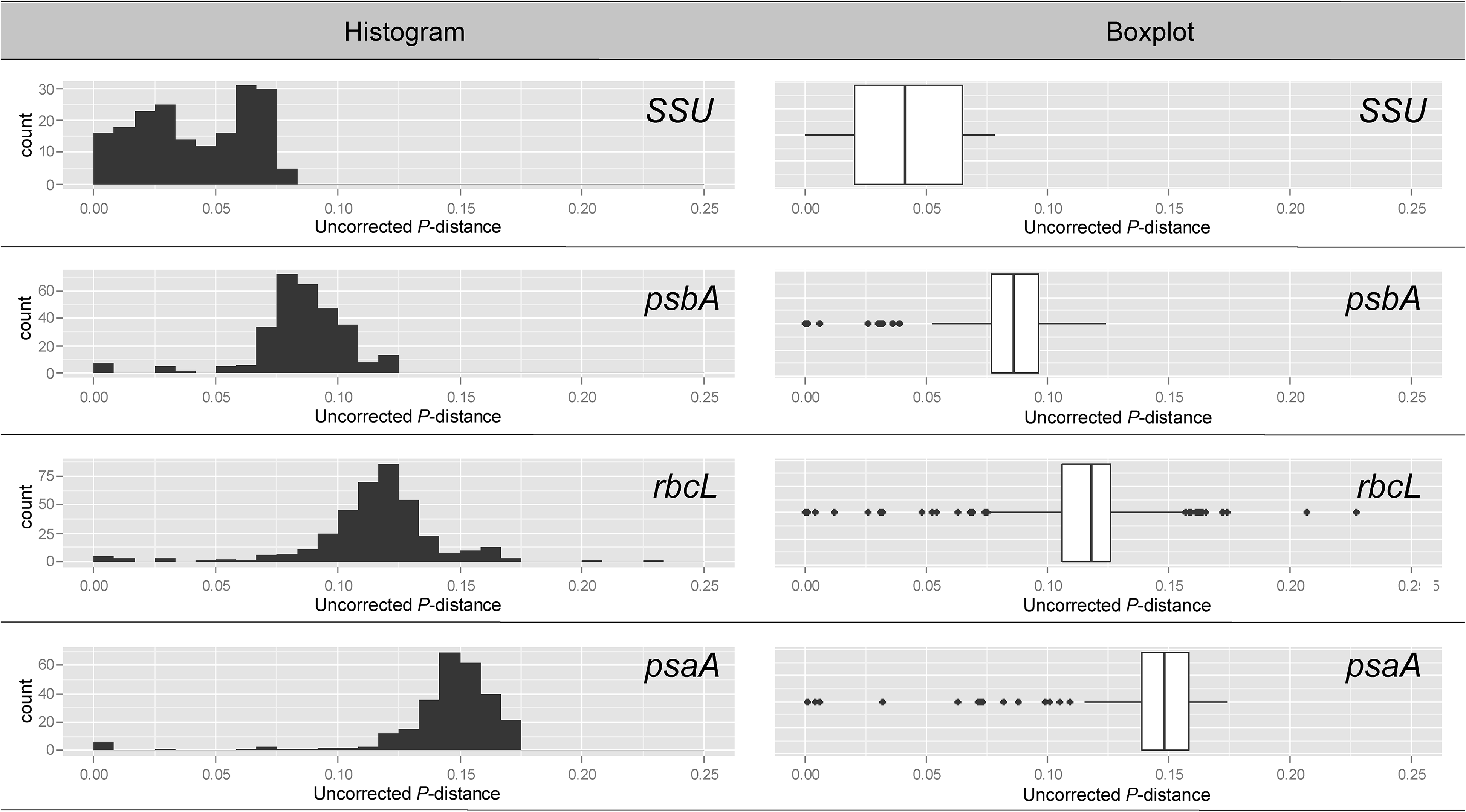

Supplement: Supplementary file 14 — Authors’ original file for figure 14 [file 40529_2012_12_MOESM14_ESM.tif]

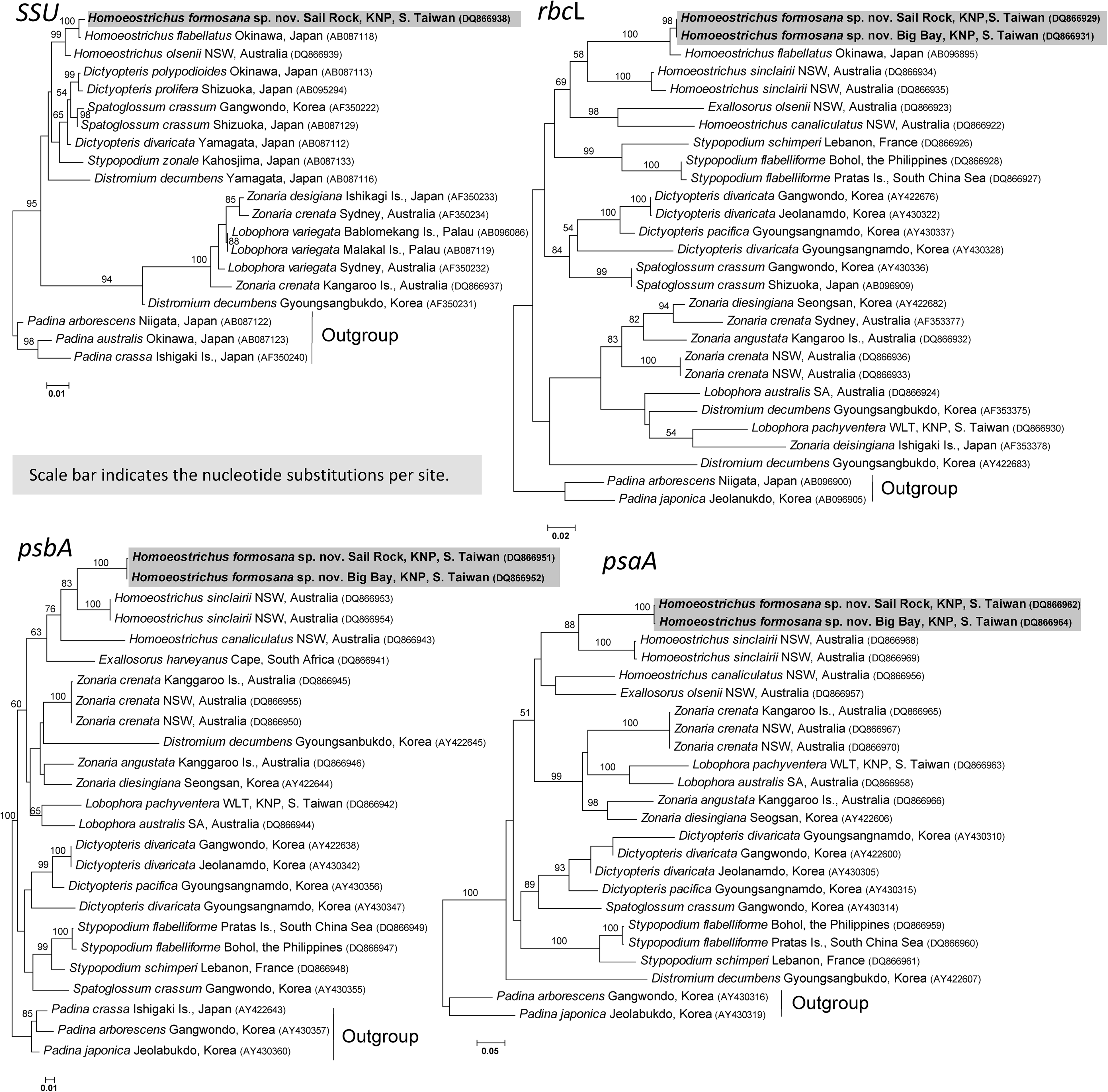

Supplement: Supplementary file 15 — Authors’ original file for figure 15 [file 40529_2012_12_MOESM15_ESM.tif]
